# Recruitment of Rec8, Pds5 and Rad61/Wapl to meiotic homolog pairing, recombination, axis formation and S-phase

**DOI:** 10.1093/nar/gkz903

**Published:** 2019-10-16

**Authors:** Soogil Hong, Jeong H Joo, Hyeseon Yun, Nancy Kleckner, Keun P Kim

**Affiliations:** 1 Department of Life Sciences, Chung-Ang University, Seoul 06974, Korea; 2 Department of Molecular and Cellular Biology, Harvard University, 52 Oxford Street, Cambridge, MA 02138, USA

## Abstract

We have explored the meiotic roles of cohesin modulators Pds5 and Rad61/Wapl, in relation to one another, and to meiotic kleisin Rec8, for homolog pairing, all physically definable steps of recombination, prophase axis length and S-phase progression, in budding yeast. We show that Pds5 promotes early steps of recombination and thus homolog pairing, and also modulates axis length, with both effects independent of a sister chromatid. [Pds5+Rec8] promotes double-strand break formation, maintains homolog bias for crossover formation and promotes S-phase progression. Oppositely, the unique role of Rad61/Wapl is to promote non-crossover recombination by releasing [Pds5+Rec8]. For this effect, Rad61/Wapl probably acts to maintain homolog bias by preventing channeling into sister interactions. Mysteriously, each analyzed molecule has one role that involves neither of the other two. Overall, the presented findings suggest that Pds5’s role in maintenance of sister chromatid cohesion during the mitotic prophase-analogous stage of G2/M is repurposed during meiosis prophase to promote interactions between homologs.

## INTRODUCTION

Meiosis is the specialized cellular program by which a diploid progenitor cell gives rise to haploid cells for gametogenesis. Accordingly, a single round of DNA replication is followed by two rounds of chromosome segregation; moreover, at the first meiotic division (MI), replicated homologous chromosomes (‘homologs’) segregate to opposite poles, a process that is absent from the mitotic program. Then at the second division (MII), sisters segregate as during mitosis (1).

During meiosis, as during the mitotic cell cycle, cohesin(s) mediate sister chromatid cohesion. However, the central unique feature of meiosis is a highly programmed sequence of interactions between homologs, and cohesins also play important roles in this process as well; in addition, cohesins are important for formation of meiotic prophase chromosome axes and for regulation of meiotic S-phase progression (2).

Meiotic recombination at the DNA level can be divided roughly into three stages (2). First, recombination is initiated by programmed double-strand breaks (DSBs) at many sites throughout the genome. Each DSB then identifies a homologous sequence on a homolog partner chromosome. Importantly, meiotic recombination occurs preferentially between homolog chromatids rather than between sister chromatids as during mitotic DSB repair. ‘Homolog bias’ is established at this very early step. Second, these initiating interactions are differentiated into two types. A few are designated to be matured as crossover (CO) products. During this process, specification of CO sites is governed by the classical process of CO interference. The majority of interactions are fated for maturation without exchange of flanking regions, i.e. as ‘non-crossovers’ (NCOs), apparently as the ‘default option’ (3). Third, after CO/NCO differentiation, both types of interactions undergo additional steps by which they are matured to their respective products.

All organisms have one or more meiosis-specific versions of the general kleisin subunit, Mcd1/Scc1/Rad21: Rec8 in virtually all organisms, in mouse, a second ortholog Rad21L, STAG3/SA3 and the SMC1 homolog SMC1 beta (4,5). However, where studied, the mitotic counterparts of these molecules also still make significant contributions (e.g. in budding yeast (6)). Cohesin-associated proteins also play important roles in meiosis, most notably the ‘cohesin gatekeeper’ Pds5/Spo76 and cohesin modulatory Rad61/Wapl. Pds5/Spo76 has been shown, in budding yeast, to be centrally important for interactions between homologs via effects on pairing and recombination, and to be less important for sister cohesion (albeit with loosening of sister axes relationships at the SC stage) (7–9). Cohesin release factor Rad61/Wapl is important for normal recombination, chromosome morphogenesis and telomere dynamics (10). In mitotic cells, Pds5 can mediate both stabilization and destabilization of cohesion (11–15) while Rad61/Wapl, which is a cohesin release factor that exerts its effects via Pds5 (12,16–18).

Roles for meiotic cohesin Rec8 in meiotic recombination have previously been defined in budding yeast. First, Rec8 plays a modest role in DSB formation and, concomitantly, is important for the immediately following resection of 5′ strand ends (19). Second, genetic analysis suggests sister recombination is promoted by cohesins and that homolog bias is ensured by the action of meiotic recombination components to counteract this cohesin-mediated channeling (20). Third, Rec8 is implicated specifically in formation of COs in the first step following CO/NCO differentiation (19), dependent upon Cdc7-mediated phosphorylation (21). Fourth, along the CO pathway, homolog bias must be actively maintained, and Rec8 is implicated as a direct mediator of this homolog bias maintenance (19). Both Pds5 and Rad61/Wapl have also been implicated in meiotic recombination in budding yeast (9,10).

Importantly, all DNA events of recombination occur in biochemical complexes that are physically associated with, and functionally dependent upon, axial chromosome structures: individual homolog axes at early stages and, at later stages, the synaptonemal complex (SC), a close-packed array of transverse filaments and other molecules that links the axes along its lengths at 100 nm distance throughout mid-late prophase (1,22). [The only exception to this rule is that the final stages of NCO formation occur in complexes that have been released from SC association (23)]. This persistent recombination complex/structure association can be rationalized, in part, by the fact that, during meiosis, recombination serves not only to shuffle genetic information between maternal and paternal genomes but also to mediate global meiosis-specific chromosome dynamics. Early axis-associated recombination interactions mediate close spatial juxtaposition of homolog axes to a certain distance, i.e. ‘pairing’ (1,19,24,25). Then, just after CO/NCO differentiation, SC formation is nucleated specifically at the sites of CO-designated interactions and, in most organisms, a subset of NCO-fated interactions (26,27). SC association is likely also important for development of CO sites into chiasmata (28).

Cohesin is universally a prominent component of the meiotic prophase chromosome structural axes (29–31). Thus, the roles of cohesins for recombination may be executed primarily or exclusively by molecules localized to these axes. Cohesin, Pds5 and Rad61/Wapl are also directly implicated in the formation of these axes, implying that they play roles for events that occur along chromosomes as well as between chromosomes, i.e. sisters and/or homologs (5,9,10,29,32).

Meiotic prophase chromosome axes are organized as linear arrays of loops, with sister loop arrays co-oriented (1,33). Since loop density is generally conserved, the numbers and lengths of the loops define the length of the axis (5,33,34). The prominence of Rec8 along axes and other considerations support the possibility that formation of meiotic loops involves direct cohesin–cohesin interactions (32) as has been discussed prominently for mitotic chromosomes in yeast (18,35) and mammalian cells (36,37). Additionally, cohesin has recently been implicated in formation of the Mb-scale loops that comprise topologically associated domains in normal mitotic G1 chromosomes (38). In budding and fission yeast meiosis, absence of either Pds5/Spo76 or Rad61/Wapl results in shorter than normal axis lengths (9,10,32), leading to the suggestion that these factors may modulating the numbers of cohesin-mediated interactions (32), has also been suggested for mitotic chromosomes (18).

It is also important to note that some of the roles of cohesins for meiotic prophase can be separated from their roles in sister chromatid cohesion. Functional studies in budding yeast have identified mutations of Rec8 that affect recombination without affecting cohesion (39). Moreover, in Coprinus, meiotic prophase cohesin axes are known to form normally, and to support SC formation, in the absence of a sister chromatid (40,41).

Cohesin has additionally been implicated in meiotic S-phase progression. Studies in budding yeast meiosis show that absence of Rec8 results in a prolongation of S-phase. Oppositely, absence of Spo11, the meiotic transesterase protein that catalyzes DSBs for initiation of recombination, results in an acceleration of S-phase progression, independent of DSBs (42). Involvement of key meiosis-specific molecules is likely related to the fact that meiotic S-phase is universally longer than mitotic S-phase. Absence of cohesin also influences S-phase progression in mitotic cells (43,44). The basis for these various effects is not clearly established, nor is it known whether the meiotic and mitotic phenomena are or are not related (‘Discussion’ section).

Here we investigate, in budding yeast, the roles of Pds5/Spo76 and Rad61/Wapl, alone and in relation to the Rec8, with respect to meiotic recombination, using physical assays for each of the steps of recombination at the DNA level. Recombination phenotypes are defined quantitatively in single and double mutant conditions at all assayable stages. We also analyze both axis/SC lengths and the cytological manifestations of COs (Zip3 foci) as well as S-phase progression and, with respect to the roles of Pds5, the processes of pairing, recombination and axis formation in the absence of replication (and, thus, a sister chromatid). We find that one or more of the three analyzed molecules play(s) an important role at every analyzable step of the recombination process, with epistasis relationships defining diverse functional interactions. New information about the meiotic recombination process also emerges along with further information regarding roles of the analyzed molecules for S-phase progression and axis formation. Notably, we show that major roles of Pds5 in recombination and axis formation are executed analogously in the presence or absence of a sister chromatid. Moreover, the observed patterns suggest specifically that Pds5’s role in maintaining cohesin for sister cohesion in the mitotic cell cycle has been repurposed during meiosis to promote the meiosis-specific program of interactions between homologs. Overall these findings shed new light on the ways in which cohesin(s) and their associated modulator molecules participate in the chromosomal events of meiosis.

## MATERIALS AND METHODS

### Strains

Detailed information on strain genotypes and characteristics can be found in Supplementary Table S1. The *HIS4LEU2* locus has been described (45).

### Meiotic time course

Yeast cells were prepared as described previously to achieve a synchronous meiotic culture (19–21). Cells were plated onto YPG agar (1% yeast extract, 2% Bacto peptone, 2% Bacto agar and 3% glycerol) and incubated at 30°C for 24 h. Single colonies were picked and inoculated in 2 ml of YPD (1% yeast extract, 2% Bacto peptone and 2% glucose) and incubated in a shaking incubator at 30°C for 24 h. To synchronize yeast cells at the G1 stage, YPD liquid cultures were transferred into supplemented pre-sporulation liquid medium (SPS; 0.5% yeast extract, 1% Bacto peptone, 0.17% yeast nitrogen base without amino acids, 0.5% ammonium sulfate, 1% potassium acetate and 50 mM potassium biphthalate, adjusted to pH 5.5 with KOH) and cultured at 30°C for 18 h. Synchronized yeast cells were harvested and washed with sporulation medium (SPM; 1% potassium acetate, 0.02% raffinose and 2 drops/l antifoam). Meiosis was induced by culturing cells in SPM medium pre-warmed at 30°C. For analysis of *PDS5-AID* cells, a single SPS culture was split and CuSO_4_ (30 μM) was added to induce expression of OsTIR1 under the control of copper-inducible CUP1 promoter. After induction of meiosis, DMSO or 2 mM auxin (3-indoleacetic acid, Sigma) was added in the culture at the indicated times. The events of meiotic divisions were monitored by fluorescence microscopy (Nikon Ti-E or Olympus BX53) after staining the nuclei with 4′,6-diamidino-2-phenylindole (DAPI).

### Physical analysis of recombination

Yeast cells sampled at each time point were resuspended in 0.1 mg/ml trioxsalen (Sigma, T6137) and exposed to 365-nm ultraviolet radiation (19–21). The cells were spheroplasted with zymolyase (US Biological, Z1004) and then lysed in guanidine-HCl solution (4.5 M guanidine-HCl, 0.1 M EDTA, 0.15 M NaCl and 0.05% sodium lauryl sarkosyl). Genomic DNA was extracted using a phenol extraction method and was treated with RNase solution (100 mM Tris, 10 mM ethylenediaminetetraacetic acid (EDTA) and 50 μg/ml RNase (Sigma, R6513)). DNA concentrations were measured using the Picogreen assay kit (Invitrogen). For 1D gel analysis, 2 μg genomic DNA was digested with 60 units of XhoI, incubated at 37°C for 3 h, precipitated with >99% ethanol and dried. The DNA samples were loaded into a 0.6% UltraKem LE agarose (Young science, Y50004) gel in 1 × TBE buffer and electrophoresed at ∼2 V/Cm for 24 h. For CO and NCO analysis, genomic DNA was digested with XhoI and NgoMIV. DNA in agarose gels was stained with 0.5 μg/ml ethidium bromide (EtBr) for 30 min. For 2D gel analysis, 2.5 μg genomic DNA was digested with XhoI and precipitated with >99% ethanol. The DNA samples were loaded into 0.4% Seakem Gold agarose (Lonza, 50152) gel in TBE buffer and electrophoresed was at ∼1 V/Cm for 21 h. The gels were stained with 0.5 μg/ml EtBr. For second-dimension electrophoresis, gel strips were placed in a 2D gel tray covered with 0.8% UltraKem LE agarose gel containing EtBr. Electrophoresis was carried out at ∼6 V/Cm at 4°C. For Southern blot analysis, hybridization was carried out using ‘Probe A’ labeled with ^32^P-dCTP radioactive nucleotides in a random primer labeling mixture (Agilent Technologies, 300392). Hybridization signals were visualized using a phosphoimage analyzer and were quantified by the Bio-Rad Quantity One software. Quantification of SEIs and dHJs signals were performed as described previously (20).

### Cohesion assay

To monitor cohesion, cells were initially grown to G1 at 30°C in SPS culture. Cells were then incubated in fresh YPD to allow cell cycle progression. Cell aliquots were resuspended in Tris-DAPI solution (10 mM Tris (pH 8.0) and 1 μg/ml DAPI) and categorized for each time point. Detailed methods for FACS and divisions have been described (42).

### Chromosome spreading and immunofluorescence

Chromosome spreads for immunofluorescence analysis were prepared as described previously (21,46,47). Briefly, cells were lysed and fixated onto a clean slide using 1% Lipsol and 3% paraformaldehyde containing 3.4% sucrose. Then, the slides were soaked in 0.2% Photo-Flo (Kodak, 146–4510), transferred to Tris-Buffered Saline (TBS) (136 mM NaCl, 3 mM KCl and 25 mM Tris–Cl, pH 8.0) and incubated for 15 min. For immunostaining, the following antibodies were used in this study: rabbit polyclonal Zip1 antibody (diluted 1:200; Santa Cruz Biotechnology, sc-33733); mouse monoclonal GFP antibody (diluted 1:5000; Santa Cruz Biotechnology, sc-9996); primary mouse monoclonal Myc antibody (diluted 1:200; Santa Cruz Biotechnology, sc-40); primary rabbit polyclonal HA antibody (diluted 1:200; Santa Cruz Biotechnology, sc-805); secondary TRITC-conjugated goat anti-rabbit lgG (diluted 1:300; Jackson ImmunoResearch, 111–025-003); secondary Alexa 488-conjugated goat anti-mouse IgG (diluted 1:300; Jackson ImmunoResearch, 115–545-003). Images were acquired using a Nikon Eclipse Ti fluorescence microscope equipped with a Nikon DS-Qi2 monochrome camera. Image deconvolution was adjusted with Nikon NIS software.

### Quantification analysis

Hybridizing DNA species were quantified using a Personal Molecular Imager system with Quantity One software from Bio-Rad. Quantification was performed as described previously (19).

### Statistical Analysis

Data for each tested single and double mutant were analyzed by Prism 5 software to give the mean ± SD (*n* ≥ 3) (solid bars in Figure 5D, E). For double mutant combinations (e.g. *pds5 rec8*, *pds5 rad61* and *rec8 rad61)*, the value of the defect *predicted* to be obtained from independent contributions of the two single mutant defects (*P*_predict_) is given by the product of the two component single mutant defects (*V*_mutant 1_$ \times$*V*_mutant 2_). For each double mutant combination, the predicted value was determined from the single mutant values from each of *n* independent experiments (*n* ≥ 3 in each case). The average value for the *n* experiments was then reported as the mean ± SD (transparent bars in Figure 5E). Statistically significant differences from multiple experiments, or between a particular double mutant and the value predicted by the hypothesis of independence (above) were assessed by Unpaired Student's *t*-tests.

## RESULTS

### Pds5 is required for early stages of meiotic recombination

**Figure 1.**
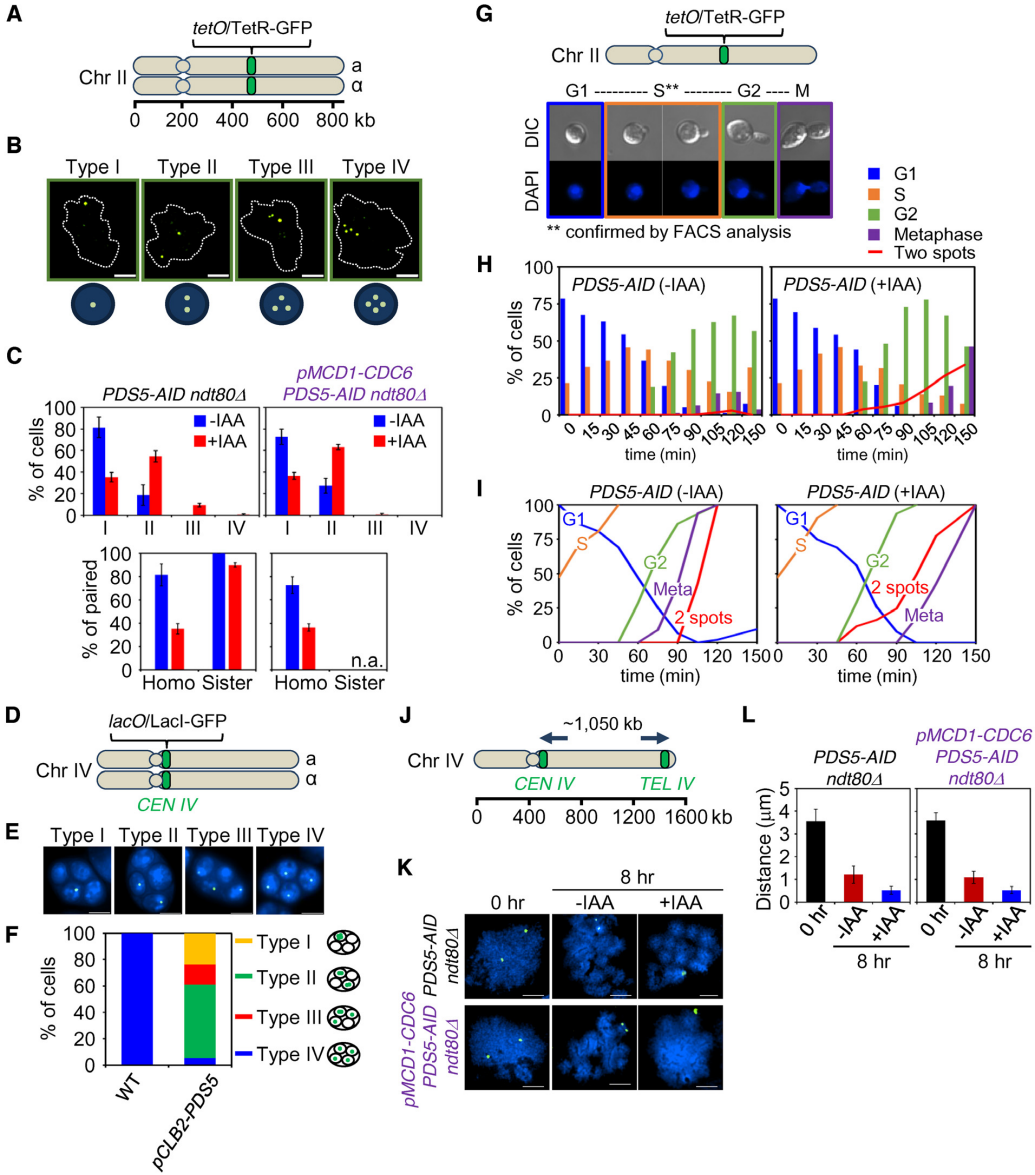
In meiotic prophase, Pds5 is specifically recruited to interhomolog (versus intersister) interactions. (**A**) Illustration of the *tet* operator (*tetO*) and Tet repressor (TetR) array. The *tet* operator arrays were integrated into chromosome II at the arm region. (**B**) Assay for sister cohesion and homolog pairing in *ndt80Δ*. Cohesion and homolog pairing activity were monitored using a diploid strain homozygous for *tetO*/TetR-GFP at chromosome II of *PDS5-AID ndt80Δ* and *pMCD1-CDC6 PDS5-AID ndt80Δ* cells that were arrested at pachytene. Cells with nuclei of type I (one focus, paired chromosomes), type II (two foci, no pairing with sister chromatid cohesion or paired chromosomes with a cohesion defect for one sister chromatid), type III (three foci, no pairing with a cohesion defect for one sister chromatid) and type IV (four foci, no pairing with cohesion defect for two pairs of sister chromatids) are shown in representative images. Chromosome spreads were prepared and stained with anti-GFP monoclonal antibody (green) and DAPI (dot lines) dyes. The scale bar indicates 2.5 μm. (**C**) Analysis of TetR-GFP focus numbers per cell. Top: The percentage of each type is shown for *PDS5-AID ndt80Δ* (*n* = 450) and *pMCD1-CDC6 PDS5-AID ndt80Δ* (*n* = 605). GFP foci were analyzed in the presence or absence of auxin in whole cells at 8 h. Bottom: Pairing of homologs and pairing (cohesion) of sister chromatids. Homo, homolog pairing; Sister, sister cohesion. Auxin (2 mM) was added to induce degradation of Pds5. Error bars represent the mean ± SD. (**D**) Illustration of the *lac* operator (*lacO*) and Lac repressor (LacI)-GFP array on *CEN4*. The *lacO* arrays were integrated into centromere area of chromosome IV. (**E**) Analysis for chromosome segregation in meiosis II. Cells with four DAPI bodies were checked for the number of LacI-GFP focus in four spores. Segregation of chromosome IV was analyzed using a strain homozygous for *CEN4-GFP*. Type I, 4:0:0:0; type II, 2:2:0:0; type III, 1:1:2:0; type IV, 1:1:1:1. (**F**) Segregation of chromosome IV in meiosis II cells. Cells with four nuclei were counted for the number of GFP focus (*lacO*/LacI-GFP signals) in a spore (*n* > 100 for WT; *n* > 250 for *pCLB2-PDS5*). The main types of chromosome segregation are illustrated in the right side of the plot. (**G**) Top: Illustration of the *tet* operator (*tetO*) and Tet repressor (TetR) array. Bottom: Representative images of cells from G1 phase to metaphase (M). (**H**) Analysis of cohesion in *PDS5-AID* cells during progression from G1 phase to metaphase. Cells were synchronized in G1, and then auxin (2 mM) was added. The percentage of cells with two GFP-foci is plotted in the presence or absence of auxin (>100 cells were counted at each time point). (**I**) Cumulative curves derived from primary data in (H). (**J**) Analysis of the axial length of chromosome IV. Chromosome IV was marked by *CEN4-GFP* and *TEL4-GFP* signals. (**K**) Representative images of *CEN4-GFP* and *TEL4-GFP* signals in *PDS5-AID ndt80Δ* and *pMCD1-CDC6 PDS5-AID ndt80Δ* cells on chromosome spreads. The scale bars indicate 2.5 μm. (**L**) Compaction analysis of meiotic chromosome in *PDS5-AID ndt80Δ* and *pMCD1-CDC6 PDS5-AID ndt80Δ* cells. The chromosome length at pachytene was determined by measuring the distance between the two GFP foci with *lacO*/LacI-GFP (*n* > 50). Auxin (2 mM) was added to induce degradation of Pds5. Error bars represent the mean ± SD.

Pds5 has previously been implicated in meiotic pairing of homologous chromosomes (‘homologs’), with modest effects on sister relationships (9). The contributions of Pds5 to pairing of homologs and pairing (cohesion) of sister chromatids can be assayed using fluorescent repressor/operator arrays at corresponding positions on homolog arms (Figure 1A). Cells were arrested at the end of prophase (using an *ndt80Δ* background) and Pds5 was severely depleted with a meiosis-specific induction of a Pds5 degron (*PDS5-AID* + IAA). The result is a severe reduction in homolog pairing but a negligible reduction in sister pairing (Figure 1B and C left; Supplementary Figure S1A–D). We also performed the analysis in a meiotic time course in a *PDS5-AIDndt80Δ tetO*/TetR-GFP heterozygous strain. A single spot, implying either no sister or paired sisters, is observed at all time points, implying that there is no loss of cohesion at any point up to late prophase *ndt80Δ* arrest, including G2 (Supplementary Figure S1D). These data confirm previous results obtained upon meiosis-specific depletion of Pds5 by expression from a mitotic-specific promoter (9) (*pCLB2-PDS5*; Supplementary Figure S1E). Robust meiotic homolog pairing is mediated largely by early stages of programmed recombination (24,48), thus implicating Pds5 in this early process (see also below).

Ultimately such recombination yields COs which, together with sister arm cohesion, provide physical connections between homologs that ensure their regular segregation to opposite poles at the first meiotic division (MI). Failure of CO formation leads to mis-segregation of homologs which, ultimately, often yields tetrads of haploid meiotic products in which two are viable and two are not. In *pCLB2-PDS5*, spore viability was significantly reduced as compared to wild-type (WT) (8 versus 99% in WT), as is the frequency of cells that carry out the first meiotic division (∼49 versus 99% in WT, as assayed at 24 h after initiation of meiosis; Supplementary Figure S2). Further, in the few asci that contain four spores, in the majority of cases, only two spores are viable (Supplementary Figure S2), pointing to a MI homolog segregation defect. Homolog mis-segregation is also shown directly by analysis of segregation of GFP-tagged centromere(s). In asci that have completed both meiotic divisions and contain four spores, WT cells exhibited normal nuclear segregation, with a single GFP signal in each spore (Type IV; Figure 1D–F; Supplementary Figure S3). However, in *pCLB2-PDS5*, 95% of asci showed aberrant segregation patterns (Types I–III; Figure 1D–F), with ∼55% of asci showing centromeres segregated into only two of the four spores (Type II; Figure 1D–F). This is the pattern expected for mis-segregation of homologs at MI. Further analysis also shows that this pattern does not result from normal MI segregation followed by a failure of sister separation at MI (Supplementary Figure S3). These findings reinforce the notion that, during meiosis, Pds5 is largely recruited to the process of interhomolog interaction.

### Pds5’s mitotic role in cohesion maintenance is executed at the stage analogous to meiotic G2/prophase

Pds5 was originally identified in a screen for cohesion maintenance at G2/M (11) and, by the one-spot/two-spot assay, depletion of Pds5 has reported to have no effect on establishment (49). It was of interest to further confirm these findings in our system. In budding yeast, mitotic G2/M is essentially analogous to mitotic prophase which, in turn, is analogous to meiotic prophase (50) and thus to the meiotic recombination period. It would be interesting to be certain that Pds5 is required for maintenance of cohesin at G2/M of the mitotic cycle and for interhomolog interactions at the analogous period (G2/prophase) of meiosis. Confirmation of previous findings in mitotic cells is also important because, despite the above findings, Pds5 has been suggested to have complex roles in both establishment and maintenance of sister cohesion (51).

To analyze the timing of cohesion absence/loss in mitotic cells, we synchronized cells at G1 (‘Materials and Methods’ section), induced a Pds5 degron during the G1/S transition (or not) and monitored progression of the two populations in parallel through mitosis by cytological assays, with confirmation by FACS analysis of DNA replication (Figure 1G and Supplementary Figure S4). Primary data (Figure 1H) were analyzed by cumulative curves that describe the percentages of cells at and after each stage, as a function of time after induction (Figure 1I). This analysis showed that, under the assayed condition, with a doubling time of ∼90 min, sister arm cohesion was normally lost ∼20 min after cells achieve metaphase whereas, when Pds5 was degraded, cohesion was lost ∼20 min before metaphase but ∼30 min after G2, demonstrating a first detectable defect in maintenance of cohesion at G2/M. We note that depletion of Pds5 also triggers a delay in occurrence of mitosis (M). This is expected because defective sister cohesion can trigger the spindle checkpoint which delays onset of anaphase.

### The roles of Pds5 for meiotic homolog pairing and for determination of chromosome axis length are executed equivalently in the presence or absence of a sister chromatid

When meiosis proceeds in the absence of DNA replication, and thus in the absence of a sister (as achieved by expression of Cdc6 from a mitosis-specific promoter), induction of the Pds5 degron confers the same severe defect in homolog pairing as seen as during otherwise normal meiosis (Figure 1C, compare right and left panels; Supplementary Figure S1A). Pds5 is also involved in determining meiotic prophase chromosome axis length, which is severely reduced when Pds5 is depleted (9). To confirm and extend this finding, we measured the distance between GFP foci to analyzed chromosome axis length after severe depletion Pds5 with meiosis-specific induction of a Pds5 degron in four conditions: at 0 h (onset of meiosis) or at 2.5 h (by which time DNA replication has been completed), both in the presence and the absence of Cdc6, in an *ndt80Δ* background to provide arrest at the end of prophase (Figure 1J–L; Supplementary Figures S5 and 6). The same significant reduction of axis lengths was observed in all cases, implying that this role of Pds5 is also exerted analogously in the presence or absence of a sister chromatid both before and after S-phase. Overall, these findings show that the meiotic role(s) of Pds5 on homolog pairing (and by extension its role(s) in meiotic recombination) and in axis length determination are equally robust when a sister chromatid is absent as in the normal case where a sister is present.

### Physical analysis of meiotic DNA recombination

Meiotic recombination proceeds in a well-defined series of steps (2). Recombination during meiosis is initiated by programmed DSB formation. After the two DSB ends undergo resection of their 5′ termini, one end identifies a homologous sequence on a partner molecule, usually on a homolog chromatid rather than the sister and establishes a nascent D-loop interaction. Thus, at this step, homolog bias is established. These interactions mediate the closer juxtaposition of homologs (generically ‘pairing’). At this point, the array of initiated interactions undergoes differentiation into two types: a few interactions are designated for eventual maturation into CO products, with positions defined by the rules of CO interference, while the remaining interactions are fated to eventually mature primarily into NCOs. Both types of interactions then mature to products via additional biochemical steps. Each CO-fated intermediate progresses first to a single-end invasion (SEI). The second DSB end is then brought into the reaction, leading to formation of a double-Holliday junction (dHJ) which, in turn, is resolved to a CO product. Along this pathway, homolog bias is actively maintained through the critical step of second end capture. Each NCO-fated intermediate is matured to the corresponding product by synthesis-dependent strand annealing. There is no information about maintenance of homolog bias during NCO formation (but see below).

**Figure 2.**
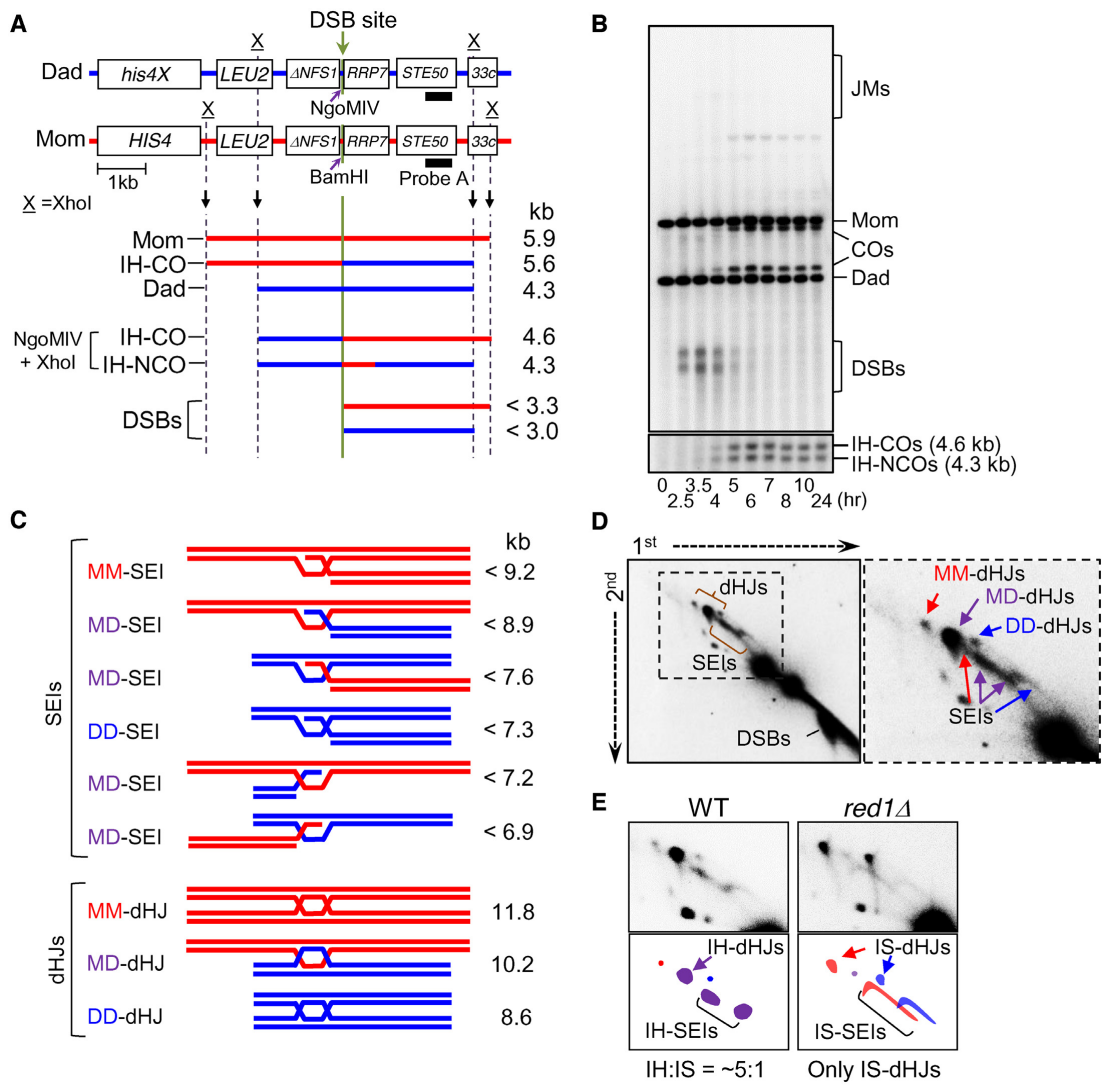
System for physical analysis of DNA events of meiotic recombination. (**A**) Physical map of the *HIS4LEU2* locus showing the DSB sites, enzyme restriction sites and the probe position for Southern blot hybridization. For physical analysis of recombination, DNA species digested with XhoI are separated on 1D or 2D gel and detected by Southern hybridization with probe A (19,20,52,69). (**B**) Representative image of 1D Southern analysis of WT. JMs, joint molecules; COs, crossovers; DSBs, double-strand breaks; IH-COs, interhomolog crossovers; IH-NCOs, interhomolog NCOs. (**C**) Diagrams of JMs, SEIs and dHJs. MM, mom–mom intersister; DD, dad–dad intersister; MD, mom–dad interhomolog. (**D**) 2D gels displaying meiotic recombination intermediates. Mom–mom IS, dad–dad IS and mom–dad IH species in red, blue and purple, respectively. (**E**) SEIs/dHJs from WT and *red1Δ* visualized with probe A. IS-SEI signals are spread out over a larger area due to the fact that the DSBs that are contained within IS-SEIs are hyperresected while IH-SEIs are identified at appropriate positions (19,20).

To further explore the roles of Pds5 for recombination, alone and in relation to Rec8 and Rad61/Wapl, and to further explore the roles of the latter molecules, we applied standard physical assays of DNA events. 1D and 2D gel analysis, combined with Southern blotting with an appropriate probe has allowed to define specific key steps in meiotic recombination as they occur over time in meiosis at a prominent DSB/recombination hotspot, *HIS4LEU2* (45,52) (Figure 2A). 1D gels define DSBs and COs or, in a different protocol, COs and NCOs (Figure 2B top, bottom). 2D gels can define extent of DSB resection (Figure 2D left; below) as well as two sequential long-lived CO-specific intermediates, SEIs and dHJs (Figure 2C and D).

It is also possible to distinguish dHJs that form between homologous non-sister chromatids (inter-homolog; ‘IH’) versus those that form between sister chromatids (inter-sister; ‘IS’) (Figure 2E). The ratio of IH-dHJs to IS-dHJs (the IH:IS dHJ ratio) defines the extent of homolog bias at that stage (the ratio is ∼5:1 in WT; Figure 2E left). A mutant defective in establishment of bias at the nascent D-loop stage (above) exhibits essentially only IS-dHJs (e.g. *red1Δ*; Figure 2E, right). Some mutants, notably including *rec8Δ*, establish homolog bias normally but fail to maintain it along the CO pathway at the SEI-to-dHJ transition, resulting in an IH:IS dHJ ratio of 1:1 (19,20).

### Rec8, Pds5 and Rad61/Wapl all play modest roles in DSB formation

**Figure 3.**
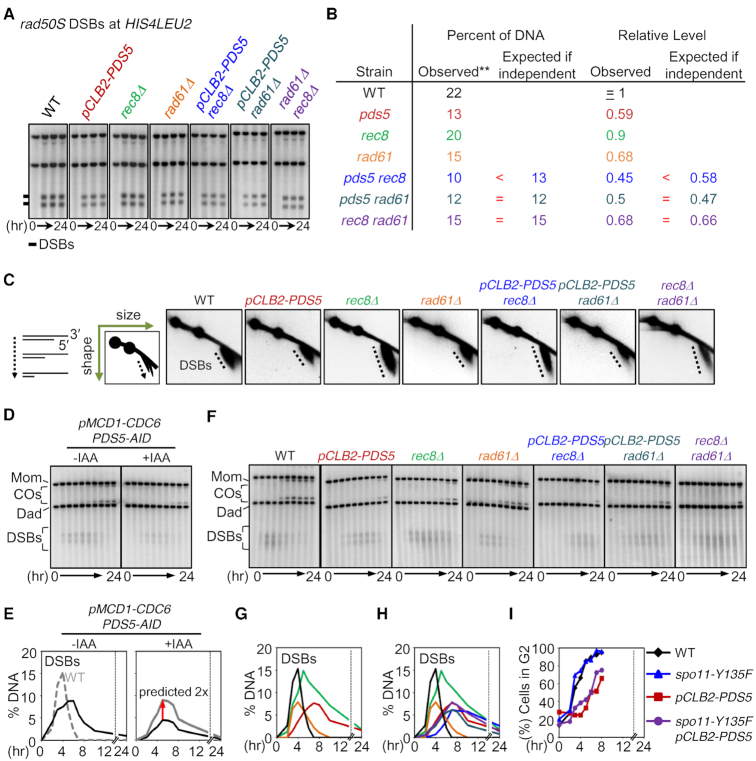
Pds5, Rad61 and Rec8 Are All Required for DSB Formation. (**A**) Analysis of *rad50S* DSBs at *HIS4LEU2*. (**B**) Quantification of DSB levels. Maximum levels of DSBs at the recombination hotspots in the *rad50S* background. The results are also in Supplementary Figure S7. (**C**) 2D gels displaying DSB resection in WT, *pCLB2-PDS5*, *rec8Δ*, *rad61Δ*, *pCLB2-PDS5 rec8Δ*, *pCLB2-PDS5 rad61Δ* and *rec8Δ rad61Δ*. (**D**) 1D Southern analysis at the *HIS4LEU2* locus for *pMCD1-CDC6 PDS5-AID* in the presence or absence of auxin. Auxin (2 mM) was added to induce degradation of Pds5. (**E**) Quantification of DSBs shown in (D). (**F**) Representative 1D Southern analysis at the *HIS4LEU2* locus in WT, *pCLB2-PDS5*, *rec8Δ*, *rad61Δ*, *pCLB2-PDS5 rec8Δ*, *pCLB2-PDS5 rad61Δ* and *rec8Δ rad61Δ*. (**G**) Quantification of DSBs in WT (black line), *pCLB2-PDS5* (dark red line), *rec8Δ* (green line) and *rad61Δ*(orange line). (**H**) Quantification of DSBs in WT (black line), *pCLB2-PDS5* (dark red line), *rec8Δ* (green line) and *rad61Δ*(orange line), *pCLB2-PDS5 rec8Δ*(blue line), *pCLB2-PDS5 rad61Δ* (dark green line) and *rec8Δ rad61Δ*(purple line). (**I**) Quantification of pre-meiotic replication progression in WT, *spo11-Y135F*, *pCLB2-PDS5* and *spo11-Y135F pCLB2-PDS5*.

Total DSB levels were conveniently assayed in a genetic background where resection and turnover are blocked (53) (e.g. *rad50S*; Figure 3A). At *HIS4LEU2*, *rec8Δ, rad61Δ* and *pCLB2-PDS5* meiotic knockdown reduced *rad50S* DSB levels to 90, 68 and 59% of the WT levels, in accord with previous studies (10,19) (Figure 3B, Supplementary Figure S7). Thus, each of the three corresponding molecules plays a modest role. Double mutant analysis revealed complex interplay among the three molecules. The role of Rad61/Wapl is independent of both the Pds5 and Rec8 roles, shown by the fact that the double mutant defect is the product of the two single mutant defects (Figure 3B and Supplementary Figure S7). In contrast, Pds5 and Rec8 have partially overlapping roles. The corresponding double mutant defect that was less than that predicted for the product of the two single mutant defects. This pattern implies that the two molecules have both a common collaborative role (e.g. as expected if Pds5 were required to stabilize Rec8) and individual independent roles.

In WT cells, DSB formation is accompanied by rapid limited resection of the 5′ strand termini at both DSB ends, as required to give 3′ single stranded tails that are coated with the RecA-homolog proteins to form nucleoprotein filaments. Nucleolytic resection of DSBs, which is mediated by Exonuclease I, can be detected in 2D gels as ∼500 nt 3′ single stranded tails in the DSB signal, due to faster migration of the single strand DNA component (19,45) (Figure 3C left). Absence of Rec8 resulted in hyper-resection, signaled by a longer tail (19) (Figure 3C). Absence of Pds5 or Rad61/Wapl had no effect on resection as seen in the corresponding single and double mutants and did not alter the hyper-resection phenotype of *rec8Δ* (Figure 3C).

### Pds5 executes its role for DSB formation in the absence of a sister chromatid

Depletion of Pds5 with a degron construct (*PDS5-AID*) conferred nearly the same defect in DSB formation as that conferred by the *pCLB2-PDS5* construct above (reductions of 50 and 40%), respectively (Supplementary Figure S7; above). A 50% reduction was observed in the absence of a sister, i.e. upon induction of degradation in *pMCD1-CDC6 PDS5-AID* (Figure 3D and E). Further, both with and without degron induction, the absolute level of DSBs observed in the *pMCD1-CDC6* background was reduced by half as compared to a WT background, in accord with the fact that there are half as many chromatids available to give rise to DSBs (Figure 3D and E, black versus gray).

### Rec8 and Pds5 (but not Rad61/Wapl) are required for timely progression through S-phase and beyond the DSB stage

In WT cells, DSBs appeared and disappeared in a timely fashion (Figure 3F–H, black). In the absence of Rec8, DSB formation timing was altered, in two respects (19) (Figure 3F–H, green). In one effect, DSBs appear later than normal, which can be attributed to delayed progression through S-phase (42). In a second effect, DSBs also persist longer than normal, which is due to defective progression of recombination beyond the DSB stage (19).

Upon depletion of Pds5, DSB appearance and progression were also both delayed (Figure 3F–H, dark red and Supplementary Figure S8). Further analysis showed that entry into G2 is delayed by 2 h in this condition (Figure 3I) and that this effect reflected a delay in progression through S-phase (Supplementary Figure S9) (analogously to the delay observed in *rec8Δ*, above). Correspondingly, abolition of DSB formation with the *spo11-Y135F* mutation had no effect on this delay of entry into G2 phase (Figure 3I). Interestingly, the *pCLB2-PDS5 rec8Δ* double mutant condition showed an even more severe delay in DSB onset than either single mutant. This might reflect partially overlapping roles of Rec8 and Pds5 for S-phase progression, analogous to the situation observed for DSBs. [We also note that elimination of DSB formation relieves the delay in meiotic divisions seen in *pCLB2-PDS5* (Supplementary Figure S2B), indicating that divisions are delayed due to checkpoint activation by unrepaired DSBs.]

In contrast, absence of Rad61/Wapl had no effect on progression into or out of the DSB stage either alone or in combination with either *rec8Δ* or *pCLB2-PDS5* (Figure 3F–H, orange, purple and dark green).

### Pds5 and Rec8 collaborate to promote maintenance of CO homolog bias, with little role for Rad61/Wapl

**Figure 4.**
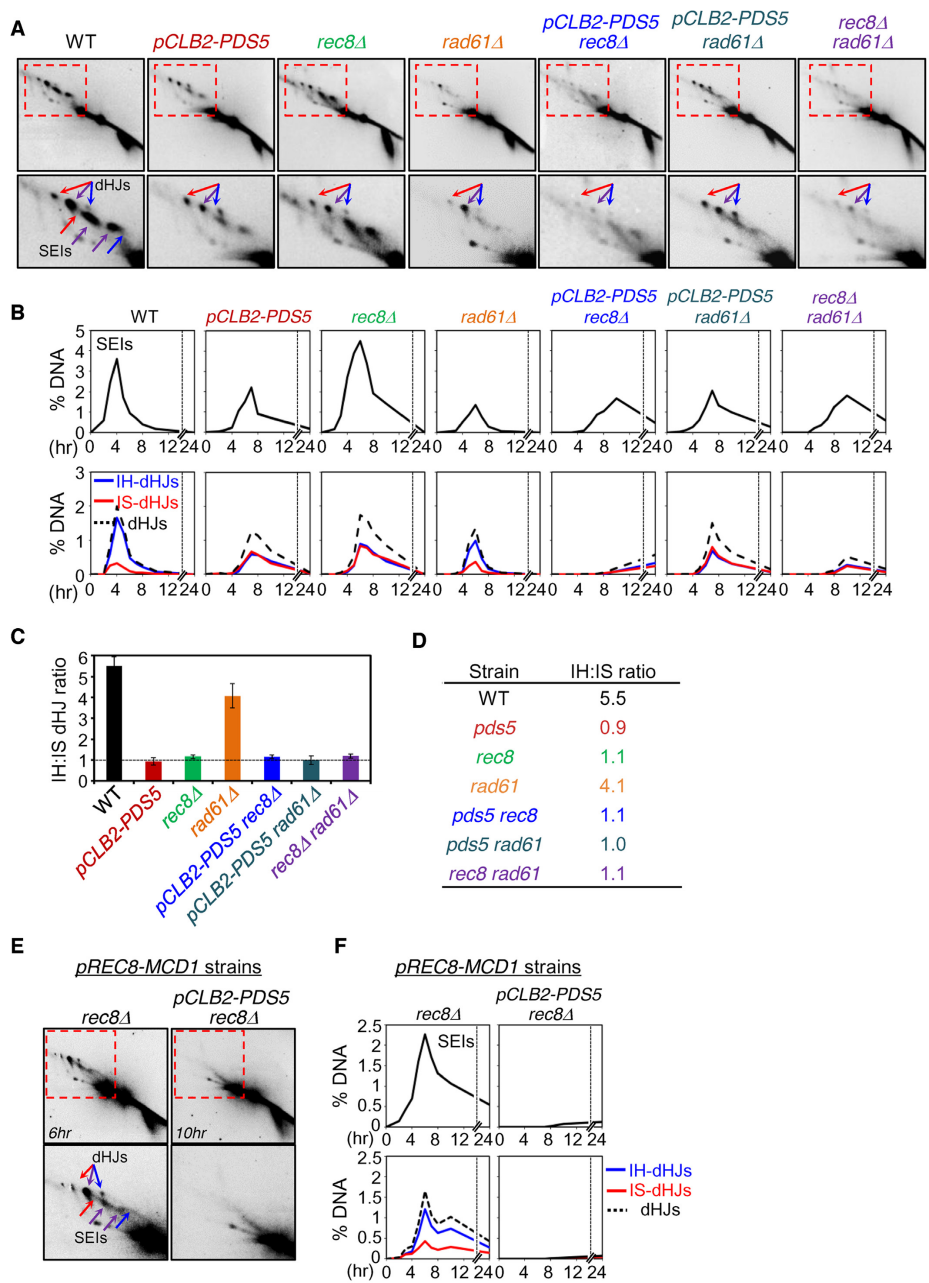
JM Formation in *pds5*, *rad61* and *rec8* Mutants. (**A**) Images of 2D gel analysis at the *HIS4LEU2* locus. Positions of SEIs and dHJs are indicated by arrows. Red arrows, mom–mom IS-JMs; blue arrows, dad–dad IS-JMs; purple arrows, mom–dad IH-JMs. (**B**) Quantification of SEIs and dHJs shown in (A). (**C**) IH:IS dHJ ratio at the *HIS4LEU2* locus in WT, *pCLB2-PDS5*, *rec8Δ*, *rad61Δ*, *pCLB2-PDS5 rec8Δ*, *pCLB2-PDS5 rad61Δ* and *rec8Δ rad61Δ*. Error bars represent the mean ± SD (*n* ≥ 3). (**D**) Summary of IH:IS dHJ ratios. (**E**) 2D gel analysis in *pREC8-MCD1 rec8Δ* and *pREC8-MCD1 pCLB2-PDS5 rec8Δ*. (**F**) Quantification of SEIs and dHJs shown in (E).

Homolog bias for meiotic COs is manifested in an IH:IS dHJ ratio of ∼5:1 as seen in a WT background (Figure 2E left, Figure 4A–D and Supplementary Figure S10) and in the *ndt80Δ* background where, in cell arrested at pachytene, dHJs accumulate rather than progressing to COs (Supplementary Figure S11). In contrast, in the absence of Rec8, the IH:IS dHJ ratio is 1:1 (19) (Figure 4A–D and Supplementary Table S2). This phenotype, also observed for certain other mutants, has previously been shown to result from a failure to maintain homolog bias at a critical intermediate step (19) (above; ‘Discussion’ section). We found that a reduction in the level of Pds5 by *pCLB2-PDS5* confers this same phenotype, which is also seen with both Rec8 and Pds5 are absent (Figure 4A–D, and Supplementary Figure S10). Thus, these two molecules collaborate to promote maintenance of CO homolog bias.

In contrast, absence of Rad61/Wapl conferred only a very small reduction in CO homolog bias (IH:IS dHJ ratio of ∼4:1; Figure 4A–D and Supplementary Table S2). However, this reduction lies in the same pathway as the [Pds5+Rec8]-promoted defect because *rec8Δ rad61Δ* and *pCLB2-PDS5 rec8Δ* double mutant strains exhibit a 1:1 IH:IS dHJ ratio (Figure 4A–D).

Interestingly, meiotic overproduction of Mcd1/Scc1 (by *pREC8-MCD1* allowing meiotic expression) can substitute for Rec8 with respect to formation of dHJs with substantial, but not perfect, homolog bias (IH:IS dHJ ratio = 3.2:1) (Figure 4E and F; Supplementary Figure S12). We further find that depletion of Pds5 in this situation (in *pREC8-MCD1 pCLB2-PDS5 rec8Δ* cells), JM levels are very low (Figure 4E and F). Given that DSBs occur at significant levels (2D gels in Figure 4E and Supplementary Figure S12), this finding points to a defect either in DSB/partner capture (as seen for Pds5 depletion in normal meiosis; ‘Discussion’ section) and/or specific designation of events to become COs.

### Rec8, Pds5 and Rad61/Wapl have distinct roles beyond DSB formation and CO homolog bias

**Figure 5.**
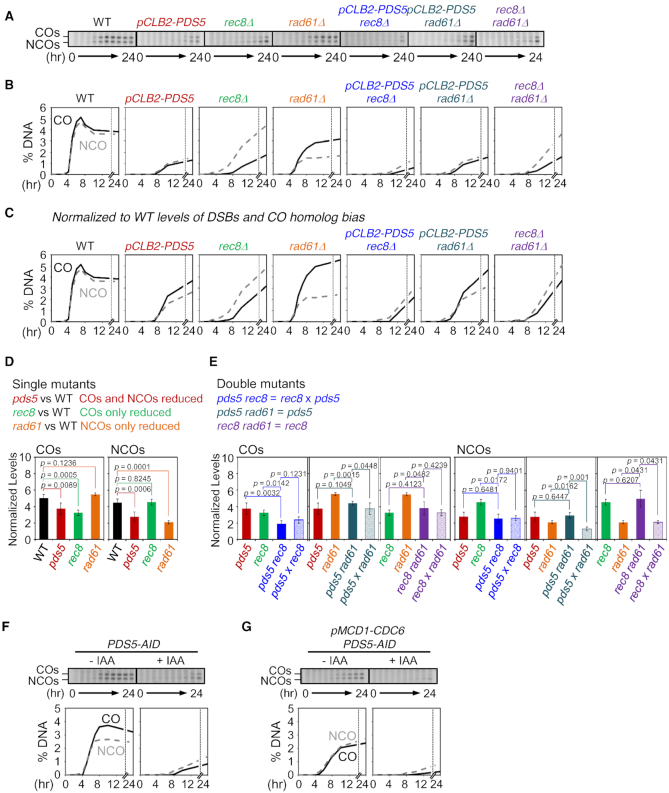
Roles of Pds5, Rad61 and Rec8 for COs and NCOs. (**A**) Gels of COs and NCOs in WT, *pCLB2-PDS5*, *rec8Δ*, *rad61Δ*, *pCLB2-PDS5 rec8Δ*, *pCLB2-PDS5 rad61Δ* and *rec8Δ rad61Δ*. (**B**) Quantification of COs and NCOs in (A). (**C**) Analysis of COs and NCOs. CO and NCO levels in mutants are normalized to WT levels of DSBs and CO homolog bias. (**D** and**E**) Mutant defects and epistatic relationships are defined by comparisons of normalized levels of COs and NCOs. (D, top) Mutant defects are defined by comparison of recombination levels of corresponding single mutants with WT. (E, top) Double mutant defects are analyzed by comparison of the double mutant defect with each of the two single mutant defects and the product of the two single mutant defects. Solid bars show mean values of normalized CO or NCO levels for the indicated mutant with error bars representing ± SD. Transparent bars in (E) are the levels predicted for independent contributions of the two component single mutant phenotypes (± SD), obtained by multiplying the values of the two single mutant defects (See ‘Materials and Methods’ section). *P*-values for all relevant comparisons are determined by Student's *t*-test. *N* ≥ 3 in all cases. See also Supplementary Table S3. (**F** and**G**) CO and NCO analysis at the *HIS4LEU2* locus for *PDS5-AID* and *pMCD1-CDC6 PDS5-AID* in the presence or absence of auxin. Auxin (2 mM) was added to induce degradation of Pds5. Quantification of COs (black lines) and NCOs (gray dashed lines) were plotted.

Absence or depletion of each of the three analyzed cohesin-related molecules conferred a significant defect in formation of COs, of NCOs or both (Figure 5A and B; Supplementary Table S3). The reductions in DSB levels and (for COs) homolog bias, described above, were contributing to these defects. To identify roles for formation of COs and NCOs beyond these two features, we normalized the observed CO and NCO to the values expected if DSBs had occurred at normal levels and (for COs) if homolog bias had been the same as in WT (Figure 5C). Analysis of these normalized values reveals additional important roles for all three molecules.

Single mutant phenotypes revealed that the three analyzed molecules have unique and distinct roles, which arise at three distinct stages in the recombination process respectively (Figure 5D). (i) *pCLB2-PDS5* coordinately reduced formation of both COs and NCOs, implying a role early in recombination, after DSBs but prior to CO/NCO differentiation. Further analysis showed that Pds5 is still required for formation of COs and NCOs in the absence of a sister, similarly as in the presence of a sister, as shown by comparing the effects of degron-induced depletion in WT and *pMCD1-CDC6* backgrounds (compare Figure 5F and G). (ii) Absence of Rec8 reduced CO formation but did not affect NCOs, implying a specific role for COs after CO/NCO differentiation. Previous analysis showed that this defect arises immediately after the designation step, with a concomitant reduction in SEIs, dHJs and COs (19). (iii) *rad61Δ* reduced NCOs and had no effect on COs. This effect must also arise after CO/NCO differentiation.

The distinct natures of these three roles were further revealed by double mutant analysis (Figure 5E).

- The *pCLB2-PDS5 rec8Δ* double mutant phenotype is the simple product of the two single mutant phenotypes. Thus, the two component defects are independent of one another. This relationship matches the fact that the two molecules act at two different steps of the recombination process (above). Moreover, these patterns imply that: (i) Rec8 has a role which is independent of Pds5; and, conversely, (ii) Pds5 has a role which is independent of Rec8.
- The effects of *rad61Δ* are unique and interesting. The single mutant strongly reduced NCOs (above). However, the *rec8Δ rad61Δ* and *pCLB2-PDS5 rad61Δ* double mutants exhibited the *rec8Δ* and *pCLB2-PDS5* single mutant phenotypes, implying that absence of Rad61 has no effect if either Rec8 or Pds5 is depleted/absent. Put the other way around, Rad61/Wapl is only required with both Rec8 and Pds5 are present. A simple interpretation of these patterns is that Rad61/Wapl promotes NCO formation by removing Rec8, whose presence requires Pds5. This, turn, is economically explained if (i) Pds5+Rec8 channels NCO-fated interactions into the sister chromatid, where they are ‘invisible’ to this analysis, and (ii) Rad61/Wapl eliminates this channeling to the sister, thus enabling formation of (interhomolog) NCOs (Discussion).

### Zip3 focus patterns mirror CO defects

**Figure 6.**
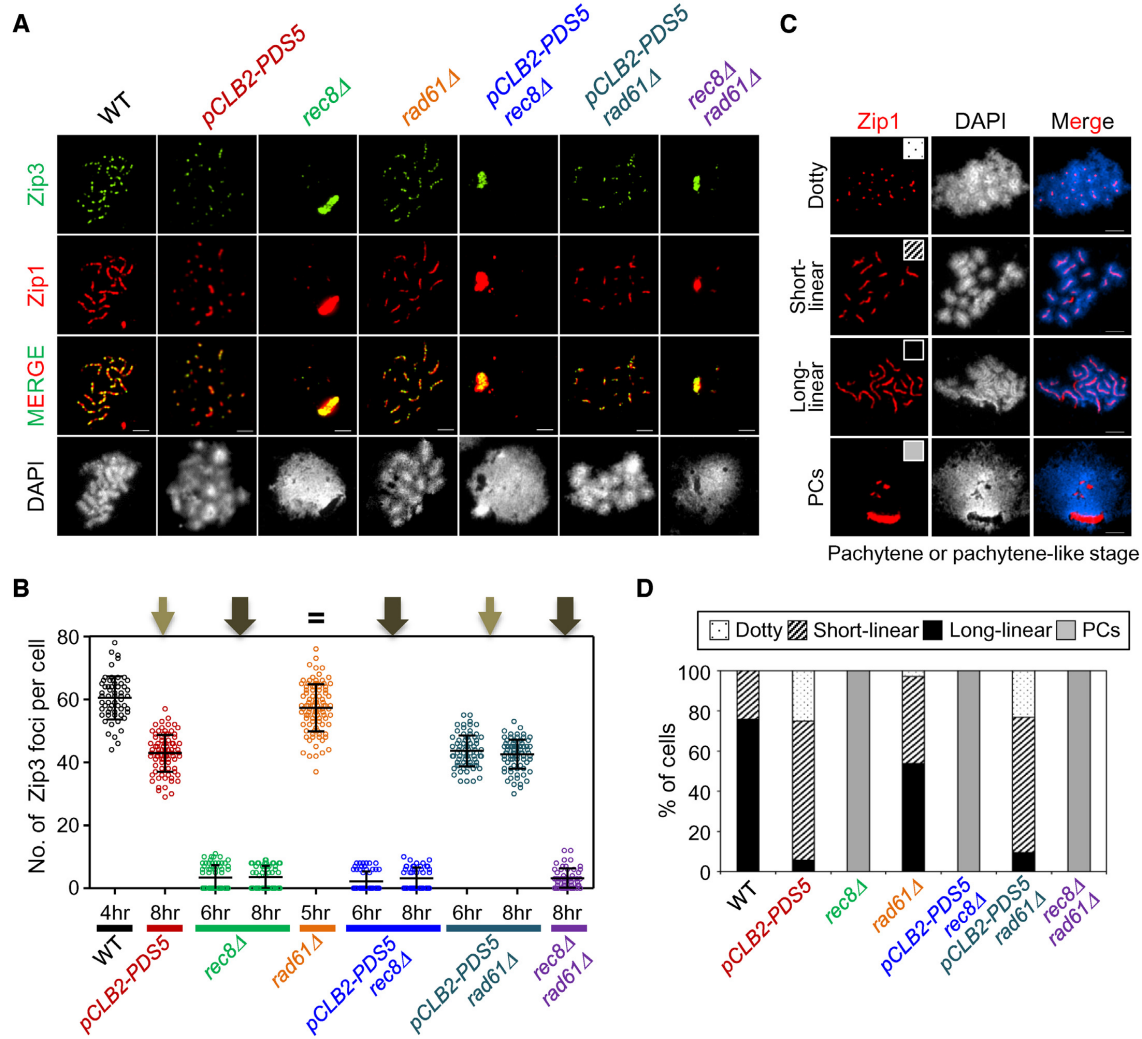
Analysis of CO-related Zip3 Focus Formation. (**A**) Localization of Zip3 together with Zip1 assembly of spread chromosomes in WT, *pCLB2-PDS5*, *rec8Δ*, *rad61Δ*, *pCLB2-PDS5 rec8Δ*, *pCLB2-PDS5 rad61Δ* and *rec8Δ rad61Δ*cells immunostained for Zip3–13myc and Zip1. Scale bars represent 2.5 μm. (**B**) Quantification of the number of Zip3 focus. The colored scatter plots show the maximum number of Zip3 focus at pachytene or pachytene-like stage (4 h for WT; 8 h for *pCLB2-PDS5*; 6 h and 8 h for *rec8Δ*; 5 h for *rad61Δ*; 6 h and 8 h for *pCLB2-PDS5 rec8Δ*; 6 h and 8 h for *pCLB2-PDS5 rad61Δ*; 8 h for *rec8Δ rad61Δ*). Error bars indicate mean ± SD (*n* = 80–150). (**C**) Representative chromosome spreads of meiotic cells immunostained for Zip1. Zip1 staining was categorized into four classes: Dotty, punctate Zip1 staining; short-linear, short Zip1-stained lines; long-linear, intensely stained Zip1 lines; PCs, polycomplexes. The scale bars indicate 2.5 μm. (**D**) Bar graph indicating the percentages of Zip1 staining types in WT and mutant strains.

Zip3 is required for progression of CO-fated interactions immediately after CO/NCO differentiation, in a transition that involves functionally coupled progression of nascent D-loops to SEIs and SC nucleation (54). This step lies upstream of the step at which CO homolog bias is maintained. Thus, Zip3 focus patterns should mirror defects in CO formation that precede this step. Effects of the analyzed mutations on Zip3 foci directly match the expectations from DNA analysis above. (i) Absence of Rec8 completely eliminated Zip3 focus formation, alone or in the presence or absence of *pCLB2-PDS5* or *rad61Δ* (Figure 6A and B). Since a small but significant number of COs form in the absence of Rec8, the absence of Zip3 foci at CO-designated sites may reflect a requirement of Zip3 focus formation for the presence of Rec8 *per se*. (ii) *pCLB2-PDS5* conferred a modest reduction in Zip3 foci, seen also in *pCLB2-PDS5 rad61Δ* (Figure 6A and B), matching the fact that absence of Pds5 modestly reduces DSBs and CO formation (as well as NCO formation) and thus Zip3 focus formation, independent of Rad61/Wapl. (iii) Absence of Rad61/Wapl had no effect on Zip3 focus numbers (Figure 6A and B), in accord with the fact that it has no role in formation of COs.

We note that the residual Zip1/Zip3 staining seen when Rec8 is absent arises from non-specific aggregates known as polycomplexes, which arise when SC formation is aberrant (additional examples in Supplementary Figure S13).

### Zip1/SC patterns mirror known effects on chromosome axis status

In WT meiosis, SC forms between the homolog axes, each of which comprises the tightly conjoined axes of its two component sister chromatids.

- In the absence of Rec8, regular axes did not form and, correspondingly, SCs were never observed, as previously described (19,29) (Figure 6A, C and D). Accordingly, also, this defect predominated in the presence or absence of *rad61Δ* or *pCLB2-PDS5*.
- *pCLB2-PDS5* exhibited very short SCs, in accord with its defect in longitudinal compaction (9) (Figure 1, 6A, C and D). Interestingly, it was previously shown that in the absence of Pds5, SCs tend to form between sister chromatids (9). That conclusion was based on immunostaining plus EM observations in an SK1 strain carrying *pCLB2-PDS5*. We confirm this tendency for the SK1 *pCLB2-PDS5* strain used in the present study, as well as in *pCLB2-PDS5 rad61Δ*. Detailed quantification identifies an average of ∼24 Zip1 dots/lines, suggesting a combination of SCs between homologs and sisters (Supplementary Figure S14). This effect could be expected if there were a looser association of sister chromatid axes. Thus, despite little or no abrogation of bulk sister cohesion in this condition by a one spot/two spot assay (9) (Figure 1), Pds5 is important for ensuring perfectly normal global cohesion of sisters.
- *rad61Δ* also exhibited many very short SCs, in accord with shortening of short SCs as previously reported for this mutant (10) (Figure 6A, C and D).
- The current study further revealed that the *pCLB2-PDS5 rad61Δ* double mutant exhibits the more severe *pCLB2-PDS5* phenotype, suggesting that Rad61/Wapl exerts its effects downstream of Pds5 (e.g. via its Pds5-dependent ability to destabilize cohesin; ‘Introduction’ section; ‘Discussion’ section).

### Epistatic interactions between *pds5*, *hos1* and *elg1*

The above results showed that the absence of Pds5 confers prominent defects in meiotic prophase I recombination, recombination-mediated homolog pairing and development of axial chromosome structure. Previous studies have characterized in detail the nature of the mitotic cell cycle and sister chromatid cohesion in *pds5* mutant strain. These studies indicate that deletion of *ELG1* suppresses the temperature sensitivity of *pds5* mutant cells (14). Further, absence of Hos1 partially suppresses the loss of G2/mitosis cohesion in *pds5* mutant cells (55). We therefore investigated whether *elg1Δ* or *hos1Δ* suppresses recombination defects caused by absence of Pds5. Analysis of meiotic recombination in *pCLB2-PDS5 elg1Δ* and *pCLB2-PDS5 hos1Δ* double mutants revealed no detectable effect of either deletion mutation on DSBs, JMs and recombinants (Supplementary Figure S15). Thus, unlike the situation in the mitotic cell cycle, neither Elg1 nor Hos1 can affect the roles of Pds5 in meiotic recombination.

## DISCUSSION

**Figure 7.**
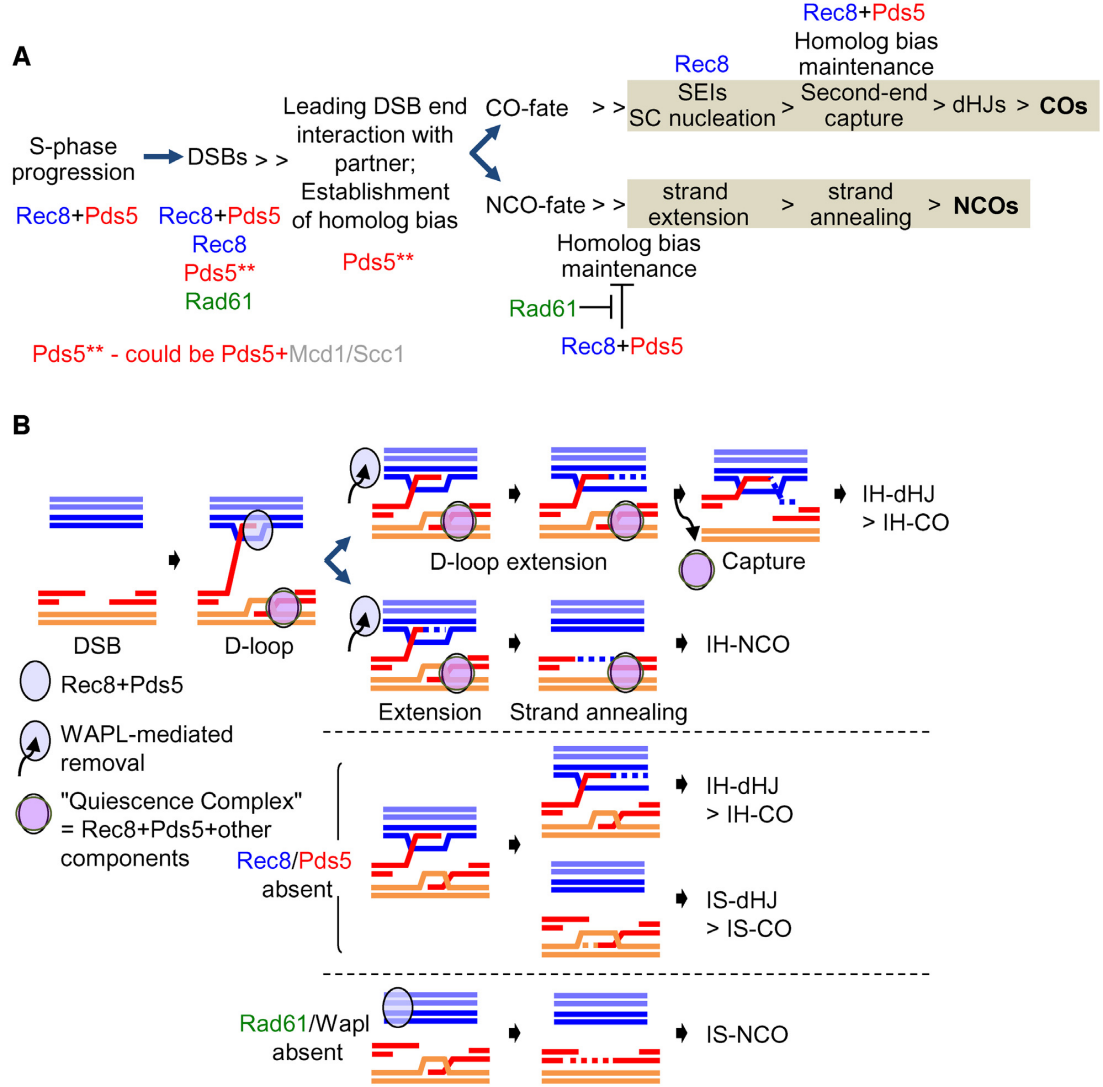
Roles of cohesin-related Molecules for Meiotic Recombination. (**A**) Scheme representing the collaborative action of Pds5, Rec8 and Rad61/Wapl in the maintenance of homolog bias for CO and NCO. The Pds5 gatekeeper collaborates with Rec8 for DSB formation and for maintenance of homolog bias, both for COs and NCOs. Rec8, Pds5 and Rad61/Wapl have distinct roles at distinct stages in the progression of DSB to CO/NCO; Rec8 is specifically required for COs at the DSB-SEI transition, as previously shown (19). By implication, Rad61/Wapl specifically promotes NCO formation by removing [Pds5+Rec8] cohesin complexes. (**B**) Homolog bias for CO and NCO during meiosis. Partner choice at the dHJ step is determined by which chromatids are engaged in the pre-dHJ. In WT meiosis, this structure forms only between non-sister chromatids and yields and IH-dHJ, and could produce an IH-CO (19,69). However, if the second DSB end is associated with the sister chromatid, and also can undergo tail extension and pre-dHJ formation, the result would be an IS-dHJ and possible, an IS-CO between sisters. In the absence of [Pds5+Rec8], first- and second-end states are now equivalent, with an equal probability of capturing another end. Rad61/Wapl is required for formation of IH-NCOs only if both Rec8 and Pds5 are present. See the ‘Discussion’ section for further details.

Previous studies have defined roles for Rec8 in meiotic chromosomal events in budding yeast, revealing participation in programmed recombination, DNA replication and axis development, as well as sister chromatid cohesion (19,21,29,56). Rec8 is the meiosis-specific analogue of the general kleisin cohesin subunit Mcd1/Scc1. Here we further investigate the roles for meiotic chromosomal events of two molecules, Pds5/Spo76 and Rad61/Wapl, that are known to modulate cohesin in mitotic cells and have been shown in previous studies to have significant roles for meiosis (7–10). Roles of, and interplay between/among Pds5, Rad61/Wapl and Rec8 with respect to chromosomal events have been defined by assessment of both single and double mutant phenotypes. Detailed analysis of meiotic recombination by physical assays of DNA events reveals that one or more of the three investigated molecules plays an important role at every assayable step of the process (Figure 7A). Roles of Pds5 for S-phase progression and interplay between Pds5 and Rad61/Wapl for axis formation are also revealed. Interesting new roles for Pds5 in an early step of recombination, and of Rad61/Wapl specific to NCO recombination, are revealed. Overall, Pds5 appears to act in all of its several roles by enforcing cohesin activity, independent of a sister chromatid for its prophase roles. These and other findings suggest that the role of this molecule defined for the prophase-analogous stage of the mitotic cell cycle has, in meiosis, been recruited to carry out diverse functions for the meiotic program of chromosomal events. The roles of Rad61/Wapl, in contrast, are more limited.

### Pds5 exerts a unique early role for recombination and, thus, homolog pairing, potentially via [Pds5+Mcd1/Scc1]

During meiotic prophase, Pds5 is required for meiotic homolog pairing and resulting segregation of homologs at MI, rather than for pairing/cohesion of sister chromatids (9). We show above that this role executed indistinguishably in the presence or absence of DNA replication and thus of a sister chromatid. During meiosis, robust pairing of homologs is dependent on DNA recombination. Accordingly, depletion of Pds5 also gives the two-viable-spore tetrad phenotype diagnostic of a defect in CO formation.

We also find that Pds5 is required both for initiation of recombination (DSBs) and for successful completion of an event that occurs after DSBs but prior to CO/NCO differentiation, both of which roles are also executed similarly in the presence or absence of a sister chromatid. In accord with the fact that homolog pairing is mediated by early steps of recombination (‘Introduction’ section), these two effects together can account for the role of Pds5 in homolog pairing. Identification of an early post-DSB role of Pds5 is unique and interesting. This is the step at which one end of each DSB searches for and identifies a homologous sequence on a homolog partner chromatid, with the resulting interaction then leading to close spatial juxtaposition of the homologs (pairing). A sister-independent defect at this stage could thus result from failure to separate one DSB end from the other end, failure to establish an appropriate contact with a partner and/or failure to achieve homolog juxtaposition.

Interestingly, also, this role of Pds5 is independent of Rec8. It is possible that Pds5 acts alone. However, we are not aware of any such role for this molecule. Thus, a more likely possibility may be that Pds5 works together with the mitotic kleisin Mcd1/Scc1, presumptively to stabilize its localization to chromosomes, in accord with the fact that NCO recombination is basically ‘mitotic recombination’ in its nature (57). This molecule is present at low levels in meiosis and is known to have significant roles, albeit poorly characterized (6). Moreover, as shown above, overproduction of Mcd1/Scc1 in meiosis can quite effectively substitute for Rec8 for events along the CO pathway. Interaction of Pds5 and Mcd1/Scc1 can also explain why some, but not all, of the roles of Pds5 for DSB formation are dependent on Rec8.

In accord with these possibilities, Mcd1/Scc1 is known to have a recombination-dependent role in ensuring clean segregation of homologs at MI (6). Also, in preliminary studies, we find that Mcd1 can be important for CO formation when Rec8 is absent (Supplementary Figure S16) and, intriguingly, that Rec8 and Mcd1 localize to distinct regions on pachytene chromosomes (Supplementary Figure S17). In addition, depletion of Pds5 dramatically decreases the number of Mcd1 foci (Supplementary Figure S17), consistent with a role for Pds5 in stabilization of Mcd1 localization.

### Homolog bias for meiotic recombination

#### [Pds5+Rec8] promotes maintenance of homolog bias for COs

Pds5 is implicated in maintenance of homolog bias along the CO pathway. For this purpose, it collaborates with Rec8. Previous studies suggest that the role of Rec8 (and thus Pds5) for maintenance of CO homolog bias comes into play at the point where SEI intermediates are extended by DNA synthesis and, then, the second DSB end is brought into the reaction (19) (Figure 7B). Absence of a ‘quiescence complex’ containing Rec8 and other molecules normally limits these interactions to the DSB end involved in forming a nascent inter-homolog D-loop and then an SEI. Instead, the process is symmetrized such that extension by synthesis can occur on either that homolog-associated DSB end or the other DSB end, which is still associated with the sister chromatid. Symmetrization of this process implies that, ultimately, the outcome is an equivalent number of either inter-homolog or inter-sister dHJs, thus explaining the 1:1 IH:IS dHJ ratio diagnostic of the CO homolog bias maintenance defect. These considerations suggest that the role of Pds5 should be to maintain Rec8-mediated sister cohesion on the DSB donor homolog.

#### Wapl counteracts [Pds5+Rec8] to actively promote maintenance of homolog bias for NCOs

One of the most unexpected findings of this study is that Wapl is dramatically and specifically required for formation of NCOs. Moreover, this defect occurs only when both Rec8 and Pds5 are present, implying that the role of Wapl is to counteract interdependent roles of Rec8/Pds5. This pattern of effects exactly matches those expected from canonical effects of the involved molecules: Pds5 acts to stabilize Rec8 localization and Wapl, acting through Pds5, releases Rec8. Pds5 stabilization of Rec8 matches the interplay observed for Pds5/Mcd1 in mitotic prophase cells (and, possibly, meiosis; above). Wapl-mediated release matches the canonical mitotic role of this molecule as a Pds5-dependent cohesin release factor (‘Introduction’ section).

In the present assay system, the NCOs detected occur between homologs and are detected by creation of diagnostic new combinations of markers from the two parents. If the corresponding interactions were diverted from the inter-homolog NCO pathway to an inter-sister pathway, e.g. to inter-sister NCOs, the resulting products would not be detected. Thus, an attractive explanation for these findings is that Wapl is required for maintenance of homolog bias for NCOs. Additionally, absence of Wapl confers only a minor defect in meiotic spore viability (10), suggesting that DSBs which normally would have become NCOs are, in fact, efficiently repaired by some alternative (invisible) pathway.

It is not a priori difficult to think that maintenance of Rec8 (cohesin) should channel a DSB interaction toward inter-sister recombination. Since the effect is after CO/NCO differentiation, it cannot be affecting DSB release or partner identification. Given the nature of the NCO assay and of the biochemical events of the NCO pathway, Wapl must exert its effect very early after CO/NCO differentiation, before the invading 3′ ssDNA end of a DSB is extended beyond the diagnostic restriction site on the homolog partner (Figure 7). For example, perhaps cohesion and/or Rec8 *per se* impedes this extension and forces the nascent inter-homolog D-loop to return to interaction with the sister chromatid. By this scenario, [Pds5+Rec8] is exerting its effects on the ‘partner’ homolog.

#### Synthesis

The above considerations suggest that, interestingly, Pds5 and Rec8 collaborate to promote homolog bias for COs and to inhibit homolog bias for NCOs. It also appears that bias is maintained for COs by an effect on the DSB donor homolog whereas bias for NCOs is maintained by an effect on the partner homolog. In both cases, Pds5 appears to be acting to stabilize Rec8 (further discussion below), positively promoting CO homolog bias and inhibiting NCO homolog bias, thus necessitating removal by Rad61/Wapl.

### The mitotic prophase role of Pds5 for cohesin stabilization is recruited to inter-homolog recombination and axis formation during meiotic prophase and to meiotic S-phase

The patterns describe above for meiotic prophase can all be explained if the role of Pds5 is to stabilize kleisin-dependent cohesin binding. For Rec8, this interplay is indicated by cases in which a *pds5* and *rec8* single mutants and a *pds5 rec8* double mutant confer the same defect. Such interplay is observed for a subset of DSBs, for homolog bias along the CO pathway, and for the inhibition of interhomolog NCO formation that is alleviated by Rad61/Wapl (e.g. for inhibition of NCO homolog bias). Analogous interplay between Pds5 and Mcd1/Scc1 is suggested underlie the early post-DSB role of Pds5. Pds5-mediated stabilization of Rec8 localization also appears to underlie both Pds5’s role in axis formation (32) and its role in meiotic S-phase progression (below). Notably, also, the two prophase roles do not require the presence of a sister chromatid.

These effects match the role of Pds5 defined in for mitotic cells of budding yeast, Pds5 is not required for stable establishment of sister cohesion in mitotic cells although it is thought to have subtler roles in maintaining a dynamic cohesion population (58) but instead is required for maintenance of cohesion after S-phase (49). Moreover, the mitotic role of Pds5 for cohesin maintenance is generally defined as occurring in ‘G2/M’, and we have been able to further pinpoint the execution point for this function to a period in between G2 and metaphase. In the mitotic program for higher eukaryotes, this period corresponds to prophase. During meiosis, the events of recombination and homolog pairing/synapsis all occur during a dramatically prolonged prophase stage that is specifically devoted to these processes. Moreover, in budding yeast mitosis, the degree of compaction achieved by M-phase (18) closely matches that observed in yeast meiotic prophase (59), suggesting that even the mitotic yeast cell cycle has prophase. Taken together these findings imply that meiosis recruits the mitotic prophase cohesin maintenance function of Pds5 to the service of meiotic prophase chromosomal events, as well as to a role in meiotic S-phase.

### Non-canonical roles for Rec8 and Rad61/Wapl

In addition to the effects described above, two mutant defects are not readily explained by canonical roles of kleisins, Pds5 or Rad61/Wapl.

- Rec8 is specifically and positively required for progression of COs immediately following CO/NCO differentiation, with resultant defects in formation of SEIs, dHJs and COs (19). We show here that this role does not require either Pds5 or Rad61/Wapl. One possibility is that, for this role, Rec8 binding to chromosomes is stabilized by meiosis-specific axis proteins, which are in intimate molecular contact with cohesins along meiotic prophase axes (60), rather than by Pds5 as in other roles ( above). We have previously also shown that formation of COs is dependent on Rec8 phosphorylation by Cdc7 (21). Phosphorylation can mediate cohesin removal by the prophase pathway. It is complicated to invoke such an effect for meiotic CO formation because it would require that Rec8 have both a positive role (defined by *rec8Δ*) and an inhibitory role that necessitates cohesin removal. However, such complexities might be accommodated within the complex framework of CO-designation and CO interference, which involve, respectively promotion and inhibition of CO formation. Also, in mammalian cells, prophase cohesin removal is mediated by Rad61/Wapl, but Rad61/Wapl is not important for CO formation. However, this is not necessarily a problem because Pds5 stabilization of Mcd1/Scc1 at mitotic G2/M in yeast does not act by protecting Mcd1/Scc1 from Rad61/Wapl but instead acts by an unknown mechanism (55,61).
- We find that Rad61/Wapl is required for DSB formation and that this role is independent of Pds5. This finding is not accommodated by current information suggesting that Wapl acts via its effects on cohesin-associated Pds5 (‘Introduction’ section), raising the possibility that this molecule might have other, unsuspected targets.

### [Pds5+Rec8] promotes meiotic S-phase progression

We previously showed that, when Rec8 is absent, progression through meiotic S-phase is delayed. Oppositely, when Spo11 transesterase, is absent, S-phase is accelerated, in an effect that does not require DSBs (42). We now show that absence of Pds5 confers the same type of defect as absence of Rec8. While we have not performed the requisite double mutant analysis, the simplest interpretation of these observations is that Pds5 and Rec8 act together to promote S-phase progression, presumably by Pds5-promoted stabilization of Rec8. We note that this effect is reminiscent of the positive effect of cohesin on S-phase progression in human cells (43) but opposite to a phenomenon described in human cells, where Pds5 acts negatively to slow replication fork progression unless neutralized by cohesin acetylation (44). This effect also differs from a Pds5 role in fork progression in mammalian cells, which is Rad21 independent (62).

We have previously suggested a hypothesis to explain these findings. Our idea emerged from considering the facts that (i) meiotic S-phase is much longer than mitotic S-phase in all studied organisms; and (ii) in budding yeast, comparison of mitotic and meiotic replication patterns suggests that, in both cases, the same replication origins are used with the same efficiencies and fork movements are the same. To reconcile these findings we suggested that the progression of S-phase is determined by regulated fork progression barriers. In this model, S-phase length would be determined by how long it takes to progress through these barriers, which in turn would be modulated in meiosis by meiosis-specific factors linked to the interhomolog interaction program. This idea motivated our investigation of the role of Spo11. Moreover, progression through a given barrier would be allowed in response to completion of requisite events behind the fork, most notably establishment of sister cohesion. This idea motivated our investigation of the role of Rec8. We then subsequently identified, in mitotic yeast cells, the existence of fork barriers, progression through which requires ATR/Mec1. While this molecule is commonly considered to be a mediator of DNA damage responses, and thus to sense aberrant situations, studies of meiosis suggest that it should be considered more generally as a coordination factor which modulates the local progression of events to ensure nucleus-wide synchrony throughout the chromosomes (63). We thus proposed that ATR/Mec1 permits progression through a given barrier in response to, e.g. establishment of sister cohesion licenses, *in trans*, initiation at a next set of origins (thus defining early and late origin firing in yeast) and, ultimately, when all forks are resolved, licensing of mitosis. In the context of this model, during meiosis, Pds5 would act to stabilize Rec8-mediated cohesion establishment behind replication forks. Observations that cohesin is enriched at replication origins in human cells (43) and Drosophila (64) are consistent with this model.

### Roles of Pds5 and Wapl for axis length determination

Yeast meiotic chromosomes comprise co-oriented linear arrays of loops. The density of loops along the axis is roughly evolutionarily conserved which, in combination with electron microscope images of meiotic chromosomes, leads to the idea of linear arrays of close-packed ‘dual loop modules’. In accord with this idea, loop lengths and axis lengths vary inversely in a variety of situations (34). Studies in many organisms identify cohesin as a major component of meiotic prophase axes and to be required for their formation (e.g. in yeast) (29). Cohesin has been suggested to be involved directly in loop formation in mitotic chromosomes, initially to explain why absence of Mcd1/Scc1 confers a defect in ‘compaction’ as well as in ‘cohesion’ (35). In meiosis, such a role is consistent with the propensity of cohesins to bind (and thus make loops at) in locally AT-rich regions (65). Other studies have shown that absence of Pds5 or of Rad61/Wapl results in hypercompaction in mitotic chromosomal rDNA (18,66) and, in meiosis, shorter prophase chromosome axes (9,10). It was thus proposed that, in meiosis, Rec8 cohesin makes loops and that absence of Pds5 results in less cohesin binding and thus longer loops and shorter axes (32).

We show here that the role of Rad61/Wapl for axis length determination is downstream of that of Pds5. This relationship matches the fact that Rad61/Wapl is thought to exert its effects through Pds5, since absence of Pds5 will eliminate any effect of Rad61/Wapl. However, absence of Pds5 is proposed to shorten axes by reducing cohesin localization (above) while absence of Rad61/Wapl, acting as a cohesin release factor, is predicted to increase cohesin localization and thus produce longer axes. It can be noted that meiotic axis length is also influenced by condensin, whose absence results in an axis length increase (67). Interplay between cohesin and condensin might open the door to additional and/or more complex roles for the involved molecules (68).

## CONCLUSION

Analysis of the roles of Rec8, Pds5 and Rad61/Wapl for meiotic chromosomal events provides further information as to how the canonical roles of these molecules have been used to promote meiosis-specific processes including programmed DNA recombination (and resultant homolog pairing), prolongation of S-phase progression and develop of meiotic prophase chromosome organization via linear loop arrays. Several identified roles do not require the presence of a sister and are thus independent of sister cohesion *per se*.

## Supplementary Material

gkz903_Supplemental_File
