# Supplementary material for: Recruitment of Rec8, Pds5 and Rad61/Wapl to meiotic homolog pairing, recombination, axis formation and S-phase
Source: Nucleic Acids Res. 2019 Oct 16;47(22):11691–708. doi: 10.1093/nar/gkz903 (PMC7145551; doi:10.1093/nar/gkz903)
Supplement: gkz903_Supplemental_File [file gkz903_supplemental_file.pdf]

SUPPLEMENTARY DATA

Recruitment of Rec8, Pds5 and Rad61/Wapl to Meiotic Homolog Pairing, Recombination, Axis Formation and S-phase

Soogil Hong,[1,†] Jeong Hwan Joo,[1,†] Hyeseon Yun,[1] Nancy Kleckner,[2,*] and Keun P. Kim[1,*]

[1]Department of Life Sciences, Chung-Ang University, Seoul 06974, Korea

[2]Department of Molecular and Cellular Biology, Harvard University, 52 Oxford Street, Cambridge, MA 02138, USA

[†] These authors contributed equally

*Correspondence: Nancy Kleckner, kleckner@fas.harvard.edu; Keun P. Kim, kpkim@cau.ac.kr

Supplementary Table S1. List of *S. cerevisiae* used in this study.

Strain†	*Genotype*‡
KKY2844	*MATa/MATα* WT/ *lys2::tetOx240::URA3,* WT/*TetR-GFP::Leu2, PDS5-AID-9myc-HygroB, pCUP1-KanMX4-OsTIR1-9myc-URA3*
KKY3843	*MATa/MATα lys2::tetOx240::URA3, TetR-GFP::Leu2, PDS5-AID-9myc-HygroB, pCUP1-KanMX4-OsTIR1-9myc-URA3, ndt80Δ::KanMX4*
KKY3903	*MATa/MATα lys2::tetOx240::URA3, TetR-GFP::Leu2, PDS5-AID-9myc-HygroB, pCUP1-KanMX4-OsTIR1-9myc-URA3, cdc6::kanMX6::pSCC1::3HA-CDC6, ndt80Δ::KanMX4*
KKY4614	*MATa/MATα trp1::hisG, leu2::LacI-GFP::KanMX4, trp1::226XlacO::NatMX*
KKY4637	*MATa/MATα trp1::hisG, leu2::LacI-GFP::KanMX4, trp1::226XlacO::NatMX, pCLB2-PDS5::KanMX4*
KKY4353	*MATa/MATα PDS5-AID-9myc-HygroB, pCUP1-KanMX4-OsTIR1-9myc-URA3, ndt80Δ::KanMX4,* WT/*leu2::LacI-GFP::KanMX4,* WT/*trp1::226XlacO::NatMX,* WT/*telIV::226XlacO::NatMX*
KKY4377	*MATa/MATα PDS5-AID-9myc-HygroB, pCUP1-KanMX4-OsTIR1-9myc-URA3, cdc6::kanMX6::pSCC1::3HA-CDC6, ndt80Δ::KanMX4,* WT/*leu2::LacI-GFP::KanMX4,* WT/*trp1::226XlacO::NatMX,* WT/*telIV::226XlacO::NatMX*
KKY885	*MATa/MATα HIS4::LEU2-(BamHI)/his4x::LEU2-(NgoMIV)--URA3, nuc1Δ::hygroB, rad50S::URA3*
KKY956	*MATa/MATα HIS4::LEU2-(BamHI)/his4x::LEU2-(NgoMIV)--URA3, nuc1Δ::hygroB, pCLB2-PDS5::KanMX4, rad50S::URA3*
KKY110	*MATa/MATα HIS4::LEU2-(BamHI)/his4x::LEU2-(NgoMIV)--URA3, nuc1Δ::hygroB, rec8Δ::KanMX4, rad50S::URA3*
KKY3737	*MATa/MATα HIS4::LEU2-(BamHI)/his4x::LEU2-(NgoMIV)--URA3, nuc1Δ::hygroB, rad61Δ::KanMX4, rad50S::URA3*
KKY1014	*MATa/MATα HIS4::LEU2-(BamHI)/his4x::LEU2-(NgoMIV)--URA3, nuc1Δ::hygroB, pCLB2-PDS5::KanMX4, rec8Δ::KanMX4, rad50S::URA3*
KKY3657	*MATa/MATα HIS4::LEU2-(BamHI)/his4x::LEU2-(NgoMIV)--URA3, nuc1Δ::hygroB, pCLB2-PDS5::KanMX4, rad61Δ::KanMX4, rad50S::URA3*
KKY3769	*MATa/MATα HIS4::LEU2-(BamHI)/his4x::LEU2-(NgoMIV)--URA3, nuc1Δ::hygroB, rec8Δ::KanMX4, rad61Δ::KanMX4, rad50S::URA3*
KKY276	*MATa/MATα HIS4::LEU2-(BamHI)/his4x::LEU2-(NgoMIV)--URA3, nuc1Δ::hygroB*
KKY2046	*MATa/MATα HIS4::LEU2-(BamHI)/his4x::LEU2-(NgoMIV)--URA3,*

	nuc1Δ::hygroB, pCLB2-PDS5::KanMX4
KKY1080	*MATa/MATα HIS4::LEU2-(BamHI)/his4x::LEU2-(NgoMIV)--URA3, nuc1Δ::hygroB, rec8Δ::KanMX4*
KKY1572	*MATa/MATα HIS4::LEU2-(BamHI)/his4x::LEU2-(NgoMIV)--URA3, nuc1Δ::hygroB, rad61Δ::KamMX4*
KKY2013	*MATa/MATα HIS4::LEU2-(BamHI)/his4x::LEU2-(NgoMIV)--URA3, nuc1Δ::hygroB, pCLB2-PDS5::KanMX4, rec8Δ::KanMX4*
KKY2328	*MATa/MATα HIS4::LEU2-(BamHI)/his4x::LEU2-(NgoMIV)--URA3, nuc1Δ::hygroB, pCLB2-PDS5::KanMX4, rad61Δ::KamMX4*
KKY3662	*MATa/MATα HIS4::LEU2-(BamHI)/his4x::LEU2-(NgoMIV)--URA3, nuc1Δ::hygroB, rec8Δ::KanMX4, rad61Δ::KamMX4*
KKY2448	*MATa/MATα HIS4::LEU2-(BamHI)/his4x::LEU2-(NgoMIV)--URA3, nuc1Δ::hygroB, PDS5-AID-9myc-HygroB, pCUP1-KanMX-OsTIR1-9myc-URA3*
KKY3020	*MATa/MATα HIS4::LEU2-(BamHI)/his4x::LEU2-(NgoMIV)--URA3, nuc1Δ::hygroB, PDS5-AID-9myc-HygroB, pCUP1-KanMX4-OsTIR1-9myc-URA3, cdc6::kanMX6::pSCC1::3HA-CDC6*
KKY3628	*MATa/MATα HIS4::LEU2-(BamHI)/his4x::LEU2-(NgoMIV)--URA3, nuc1Δ::hygroB, spo11(Y135F)-HA::URA3*
KKY3626	*MATa/MATα HIS4::LEU2-(BamHI)/his4x::LEU2-(NgoMIV)--URA3, nuc1Δ::hygroB, pCLB2-PDS5::KanMX4, spo11(Y135F)-HA::URA3*
KY3969	*MATa/MATα HIS4::LEU2-(BamHI)/his4x::LEU2-(NgoMIV)--URA3, nuc1Δ::hygroB, pREC8-MCD1-HA3::LEU2-rec8Δ::KanMX4*
KKY3981	*MATa/MATα HIS4::LEU2-(BamHI)/his4x::LEU2-(NgoMIV)--URA3, nuc1Δ::hygroB, pREC8-MCD1-HA3::LEU2-rec8Δ::KanMX4, pCLB2-PDS5::KanMX4*
KKY2029	*MATa/MATα HIS4::LEU2-(BamHI)/his4x::LEU2-(NgoMIV)--URA3, nuc1Δ::hygroB, ZIP3-13myc::HygroB/Zip3*
KKY2550	*MATa/MATα pCLB2-PDS5::KanMX4, Zip3/ZIP3-13myc::HygroB*
KKY2030	*MATa/MATα HIS4::LEU2-(BamHI)/his4x::LEU2-(NgoMIV)--URA3, nuc1Δ::hygroB, rec8Δ::KanMX4, ZIP3-13myc::HygroB/Zip3*
KKY3762	*MATa/MATα rad61Δ::KamMX4, Zip3/ZIP3-13myc::HygroB*
KKY2916	*MATa/MATα HIS4::LEU2-(BamHI)/W*T*, nuc1Δ::hygroB, pCLB2-PDS5::KanMX4, rec8Δ::KanMX4, ZIP3-13myc::HygroB/Zip3*
KKY3670	*MATa/MATα pCLB2-PDS5::KanMX4, rad61Δ::KamMX4, Zip3/ZIP3-13myc::HygroB*
KKY4224	*MATa/MATα HIS4::LEU2-(BamHI)/his4x::LEU2-(NgoMIV)--URA3,*

	nuc1Δ::hygroB, rec8Δ::KanMX4, rad61Δ::KamMX4, ZIP3-13myc::HygroB/Zip3
KKY3061	*MATa/MATα lys2::tetOx240::URA3, TetR-GFP::Leu2, ndt80Δ::KanMX4*
KKY3024	*MATa/MATα lys2::tetOx240::URA3, TetR-GFP::Leu2, pCLB2-PDS5::KanMX4, ndt80Δ::KanMX4*
KKY389	*MATa/MATα HIS4::LEU2-(BamHI)/his4x::LEU2-(NgoMIV)--URA3, nuc1Δ::hygroB, ndt80Δ::KanMX4*
KKY2159	*MATa/MATα HIS4::LEU2-(BamHI)/his4x::LEU2-(NgoMIV)--URA3, nuc1Δ::hygroB, pCLB2-PDS5::KanMX4, ndt80Δ::KanMX4*
KKY2136	*MATa/MATα HIS4::LEU2-(BamHI)/his4x::LEU2-(NgoMIV)--URA3, nuc1Δ::hygroB, hos1Δ::KanMX4*
KKY2410	*MATa/MATα HIS4::LEU2-(BamHI)/his4x::LEU2-(NgoMIV)--URA3, nuc1Δ::hygroB, pCLB2-PDS5::KanMX4, hos1Δ::KanMX4*
KKY1048	*MATa/MATα HIS4::LEU2-(BamHI)/his4x::LEU2-(NgoMIV)--URA3, nuc1Δ::hygroB, elg1Δ::KanMX4*
KKY1112	*MATa/MATα HIS4::LEU2-(BamHI)/his4x::LEU2-(NgoMIV)--URA3, nuc1Δ::hygroB, pCLB2-PDS5::KanMX4, elg1Δ::KanMX4*
KKY5191	*MATa/MATα trp1::hisG, leu2::LacI-GFP::KanMX4/*WT*, TRP1::226XlacO::Clonat/*WT
KKY5188	*MATa/MATα trp1::hisG, leu2::LacI-GFP::KanMX4/*WT*, TRP1::226XlacO::Clonat/*WT, *pCLB2-PDS5::KanMX4*
KKY5184	*MATa/MATα* WT*/lys2::tetOx240::URA3,* WT*/TetR-GFP::Leu2, PDS5-AID-9myc-HygroB, pCUP1-KanMX4-OsTIR1-9myc-URA3, ndt80Δ::KanMX4*
KKY5186	*MATa/MATα* WT*/lys2::tetOx240::URA3,* WT*/TetR-GFP::Leu2, PDS5-AID-9myc-HygroB, pCUP1-KanMX4-OsTIR1-9myc-URA3, cdc6::kanMX6::pSCC1::3HA-CDC6, ndt80Δ::KanMX4*
KKY5164	*MATa/MATα* WT*/PDS5-9myc::KanMX,*
KKY5193	*MATa/MATα* WT*/PDS5-9myc::KanMX, rec8Δ::KamMX4*
KKY5195	*MATa/MATα* WT*/PDS5-9myc::KanMX, rad61Δ::KamMX4*
KKY5197	*MATa/MATα* WT*/PDS5-9myc::KanMX, rec8Δ::KamMX4, rad61Δ::KamMX4*
KKY5244	*MATa/MATα Mcd1-9myc-HygroB/*WT*, REC8-3HA::URA3/*WT
KKY5250	*MATa/MATα Mcd1-9myc-HygroB/*WT*, REC8-3HA::URA3/*WT, *pCLB2-PDS5::KanMX4*
KKY4766	*MATa/MATα HIS4::LEU2-(BamHI)/his4x::LEU2-(NgoMIV)--URA3, pCLB2-MCD1::KanMX4*
KKY5207	*MATa/MATα HIS4::LEU2-(BamHI)/his4x::LEU2-(NgoMIV)--URA3, nuc1Δ::hygroB, pCLB2-MCD1::KanMX4, rec8Δ::KanMX4*

† All strains are isogenic derivatives of SK1 background.

‡ All strains are also homozygous for the mutation *ho::hisG*, *leu2::hisG*, *ura3 (ΔPstI-SmaI)*.

Supplementary Table S2. Summary of mutant phenotypes in meiotic recombination.

Genotypes	*rad50S* DSB levels	Relative *rad50S* DSB level	SD in DSB levels	*CO: NCO levels	DSB resection	DSB timing	Post-DSB progression	IH:IS dHJ ratio
WT	22	= 1	3.56 (n = 3)	5.0 4.7	= WT; normal	= WT	= WT	5.5 ± 0.4:1 (n = 4)
pCLB2-PDS5	13	~ 0.59	1.33 (n = 3)	1.3 1.57	= WT	delay	delay	0.9 ± 0.2:1 (n = 3)
rec8Δ	20	~ 0.9	2.15 (n = 3)	1.74 4.39	extensive	= WT	delay	1.1 ± 0.1:1 (n = 3)
rad61Δ	15	~ 0.68	1.35 (n = 3)	3.16 1.68	= WT	= WT	= WT	4.1 ± 0.6:1 (n = 3)
pCLB2-PDS5 rec8Δ	10	~ 0.45	3.26 (n = 3)	0.61 1.37	extensive	delay	delay	1.1 ± 0.2:1 (n = 3)
pCLB2-PDS5 rad61Δ	12	~ 0.5	2.54 (n = 3)	1.55 1.78	= WT	delay	delay	1.0 ± 0.2:1 (n = 3)
rec8Δ rad61Δ	15	~ 0.68	1.43 (n = 3)	1.59 3.65	extensive	= WT	delay	1.1 ± 0.1 :1 (n = 3)

*CO/NCO levels in Figure 5B.

Supplementary Table S3. CO and NCO formation.

Strains	*CO	*NCO
WT	5.0 ± 0.5 (n = 5)	4.5 ± 0.4 (n = 5)
pCLB2-PDS5	1.4 ± 0.3 (n = 6)	1.7 ± 0.3 (n = 6)
rec8Δ	1.8 ± 0.2 (n = 4)	4.1 ± 0.3 (n = 4)
pCLB2-PDS5 rec8Δ	0.5 ± 0.1 (n = 3)	1.2 ± 0.2 (n = 3)
rad61Δ	3.1 ± 0.1 (n = 3)	1.5 ± 0.2 (n = 3)
pCLB2-PDS5 rad61Δ	1.6 ± 0.1 (n = 4)	1.8 ± 0.2 (n = 4)
rec8Δ rad61Δ	1.6 ± 0.3 (n = 3)	3.5 ± 0.7 (n = 3)

*Data represent mean ± SD.

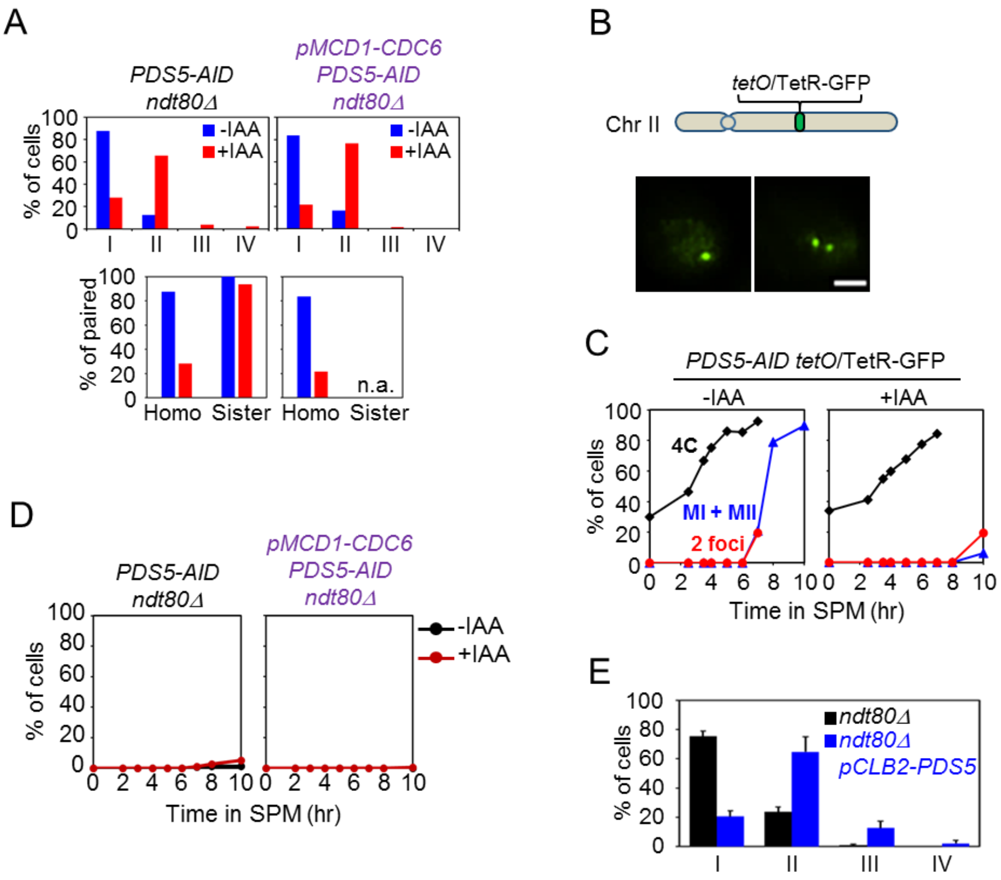

Supplementary Figure S1. *tetO*/TetR-GFP assay for sister cohesion and homolog pairing.

(**A**) Cohesion and homolog pairing activity were monitored using a diploid strain homozygous for *tetO*/TetR-GFP at chromosome II of *PDS5-AID ndt80Δ* and *pMCD1-CDC6 PDS5-AID ndt80Δ* cells that were arrested at pachytene. Cells with nuclei of type I (one focus, paired chromosomes), type II (two foci, no pairing with sister chromatid cohesion or paired chromosomes with a cohesion defect for one sister chromatid), type III (three foci, no pairing with a cohesion defect for one sister chromatid), and type IV (four foci, no pairing with cohesion defect for two pairs of sister chromatids) are shown in representative images in Figure 1B. Analysis of TetR-GFP focus numbers per cell. Top: The percentage of each type is shown for *PDS5-AID ndt80Δ* (n = 129) and *pMCD1-CDC6 PDS5-AID ndt80Δ* (n = 129). GFP foci were analyzed in the presence or absence of auxin in whole cells at 8 h. Bottom: Pairing of homologs and pairing (cohesion) of sister chromatids. Homo, homolog pairing; Sister, sister cohesion. Auxin (2 mM) was added to induce degradation of Pds5 at 2.5 hr. (**B**) Illustration of the *tet* operator (*tet*O) and Tet repressor (TetR) array system. The tet operator arrays were integrated into

chromosome II at the arm region. *tetO*/TetR-GFP signals were visualized by fluorescence microscopy. One focus indicates replicated, but non-separated sister; two GFP foci indicates replicated and separated sister. (**C**) Percentages of cells in meiotic cultures showing pre-meiotic replication (black), meiotic division (blue), and two GFP foci (red) in WT and *PDS5-AID tetO*/TetR-GFP cells. Auxin (2 mM) was added to induce degradation of Pds5. (**D**) Percentages of cells in meiotic cultures showing two GFP foci in *PDS5-AID ndt80Δ* and *pMCD1-CDC6 PDS5-AID ndt80Δ* cells. Auxin (2 mM) was added to induce degradation of Pds5 at 2.5 hr. (**E**) Analysis of TetR-GFP focus formation. The percentage of each type is shown for *ndt80Δ* (n = 147) and *ndt80Δ pCLB2-PDS5* (n = 212).

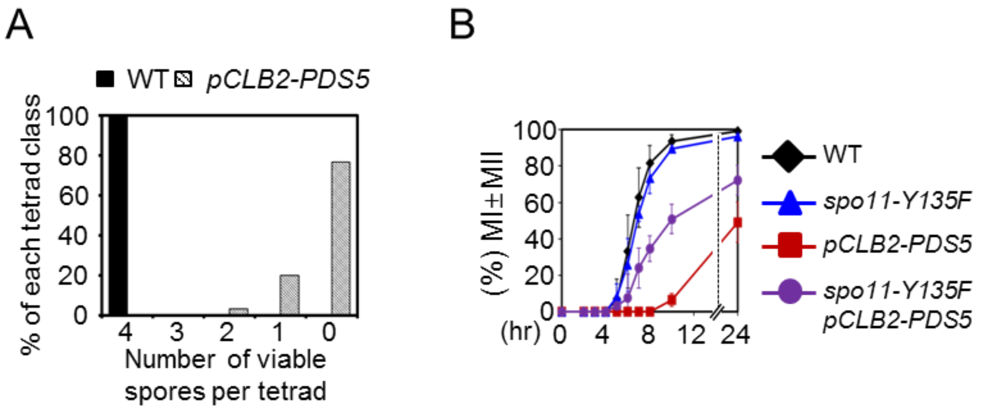

Supplementary Figure S2. Spore viability and meiotic division in WT and *pCLB2-PDS5* cells.

(**A**) WT and *pCLB2-PDS5* cells were sporulated on SPM medium and the number of viable spores was determined. (**B**) Meiotic nuclear division in WT, *spo11-Y135F*, *pCLB2-PDS5* and *spo11-Y135F pCLB2-PDS5*. Cells were sporulated and then assessed for the formation of nuclei. Approximately 200 cells were evaluated for meiotic division at each time point.

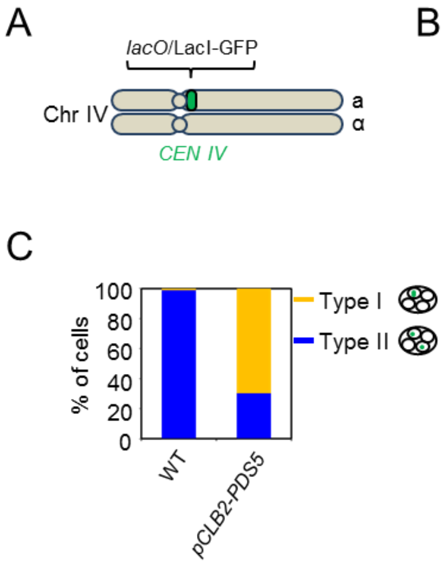

Supplementary Figure S3. Analysis of sister segregation in WT and *pCLB2-PDS5* cells.

(**A**) Illustration of the lac operator (*lacO*) and Lac repressor (LacI)-GFP array on *CEN4*. The *lacO* arrays were integrated into centromere area of chromosome IV. (**B**) Cells with four DAPI bodies were checked for the number of LacI-GFP focus in four spores. Segregation of chromosome IV was analyzed using a strain heterozygous for *CEN4-GFP*. Type I, 2:0:0:0; Type II, 1:1:0:0. The scale bars indicate 2.5 μm. (**C**) Segregation of chromosome IV in meiosis II cells. Cells with four nuclei were counted for the number of GFP focus (*lacO*/LacI-GFP signals) in a spore (n > 230 for WT, n > 205 for *pCLB2-PDS5*). The main types of chromosome segregation are illustrated in the right side of the plot.

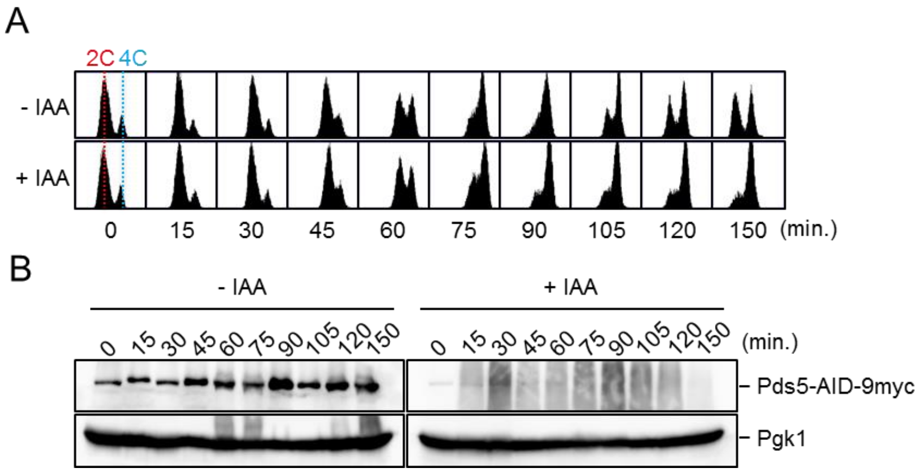

Supplementary Figure S4. Analysis of replication timing and protein degradation in the presence or absence of Pds5.

(**A**) DNA contents of *PDS5-AID* cells in the presence or absence of auxin. (**B**) Analysis of Pds5 protein degradation induced by auxin-degron. Cells were subjected to Western blot using anti-Myc and anti-Pgk1 antibodies.

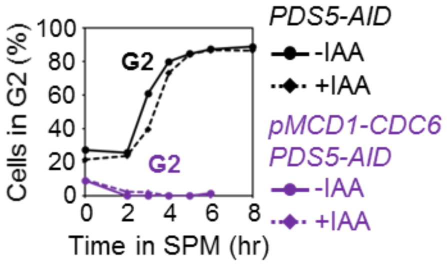

Supplementary Figure S5. Analysis of replication timing in the presence or absence of sister.

Quantification of pre-meiotic replication progression in *PDS5-AID* and *pMCD1-CDC6 PDS5-AID* cells. DNA replication was measured via flow cytometry analysis for each strain.

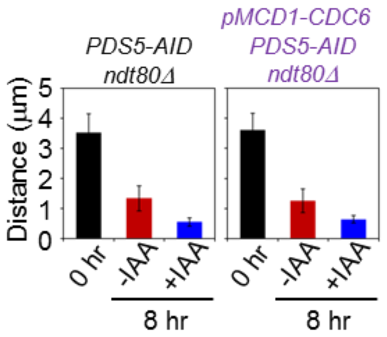

Supplementary Figure S6. Analysis of chromosome compaction in Pds5 depletion after S phase in the presence or absence of sister.

Chromosome IV was marked by *CEN4-GFP* and *TEL4-GFP* signals are shown in Figure 1J. Compaction analysis of meiotic chromosome length at pachytene was determined by measuring the distance between the two foci with *lac*O/LacI-GFP (n > 40). Auxin (2 mM) was added to induce degradation of Pds5 after S phase. Error bars represent the mean ± SD.

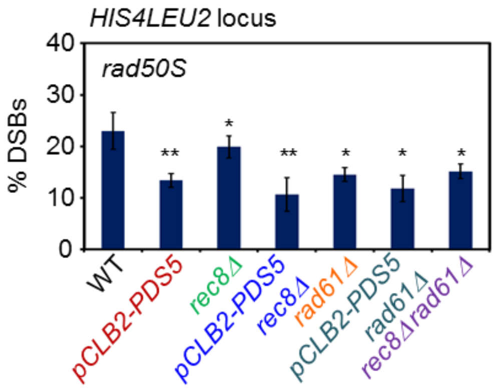

Supplementary Figure S7. DSB levels in *rad50S*.

Quantification of DSB levels. Maximum levels of DSBs at the recombination hotspots in the *rad50S* background. Error bars indicate the mean ± SD (n = 3). Asterisks represent significant differences (*P < 0.5, **P < 0.01, and ***P < 0.001; Student's t test) relative to the wild type.

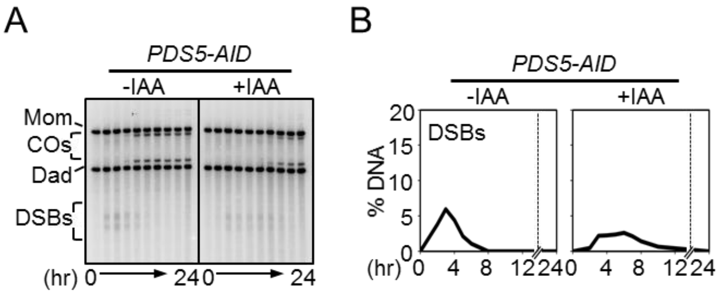

Supplementary Figure S8. Pds5 plays important roles in DSB formation.
(**A**) 1D gel analysis at the *HIS4LEU2* locus for *PDS5-AID* in the presence or absence of auxin. Auxin (2 mM) was added to induce degradation of Pds5 of sporulation medium (SPM) culture. (**B**) Quantification of DSBs shown in (A).

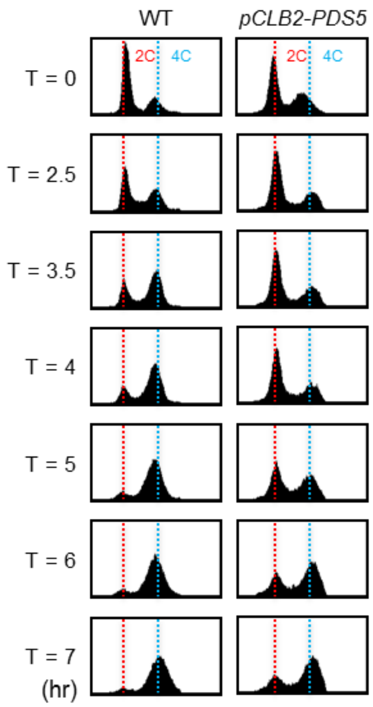

Supplementary Figure S9. Pre-meiotic replication in WT and *pCLB2-PDS5*. Flow cytometry analysis of cell cycle progression of WT and *pCLB2-PDS5* during synchronous meiosis.

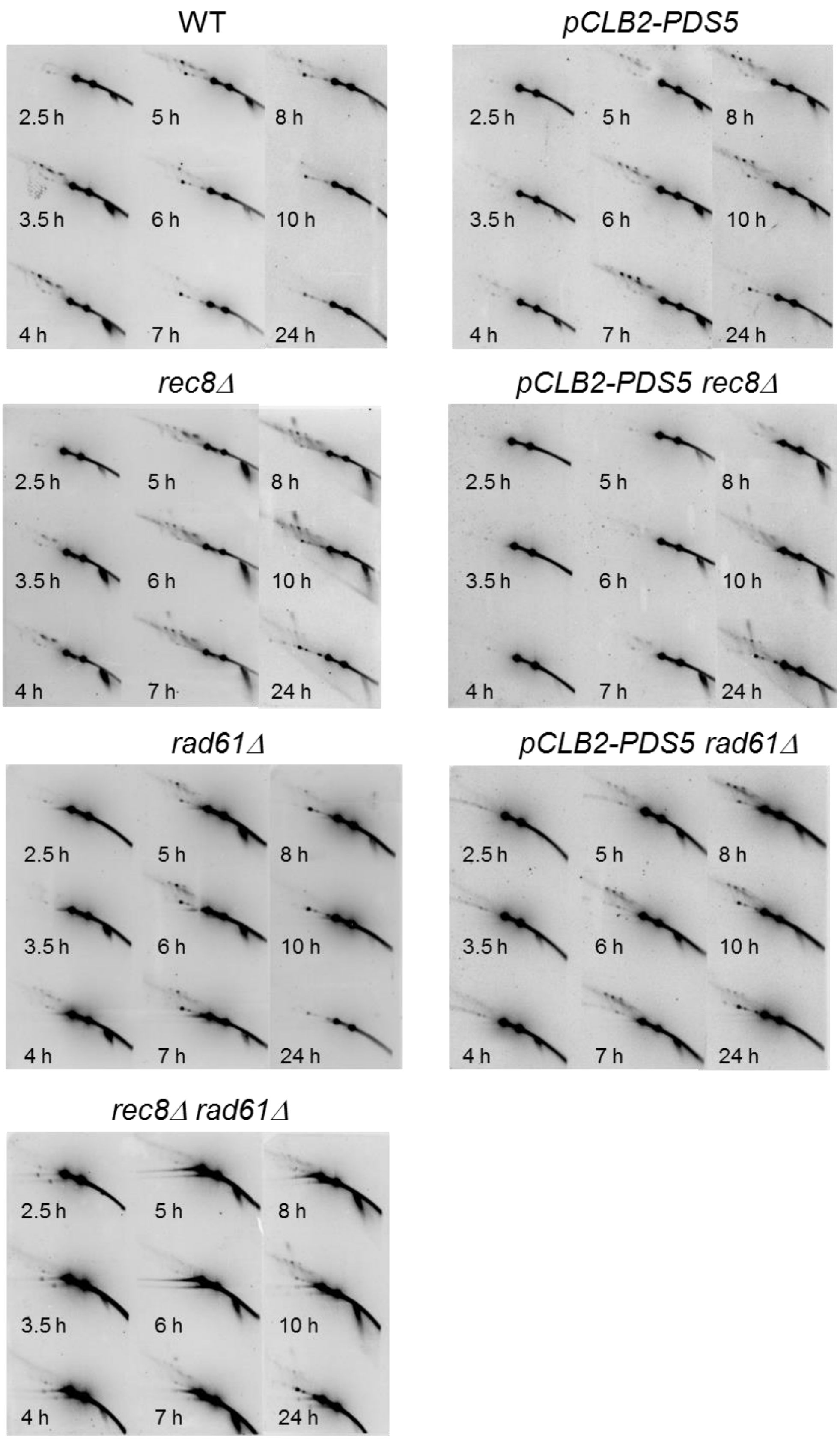
WT
2.5 h
5 h
8 h
3.5 h
6 h
10 h
4 h
7 h
24 h
pCLB2-PDS5
2.5 h
5 h
8 h
3.5 h
6 h
10 h
4 h
7 h
24 h
rec8Δ
2.5 h
5 h
8 h
3.5 h
6 h
10 h
4 h
7 h
24 h
pCLB2-PDS5 rec8Δ
2.5 h
5 h
8 h
3.5 h
6 h
10 h
4 h
7 h
24 h
rad61Δ
2.5 h
5 h
8 h
3.5 h
6 h
10 h
4 h
7 h
24 h
pCLB2-PDS5 rad61Δ
2.5 h
5 h
8 h
3.5 h
6 h
10 h
4 h
7 h
24 h
rec8Δ rad61Δ
2.5 h
5 h
8 h
3.5 h
6 h
10 h
4 h
7 h
24 h

Supplementary Figure S10. 2D gel analysis of WT and mutants.

Representative 2D gel images showing recombination intermediate species at each indicated time point.

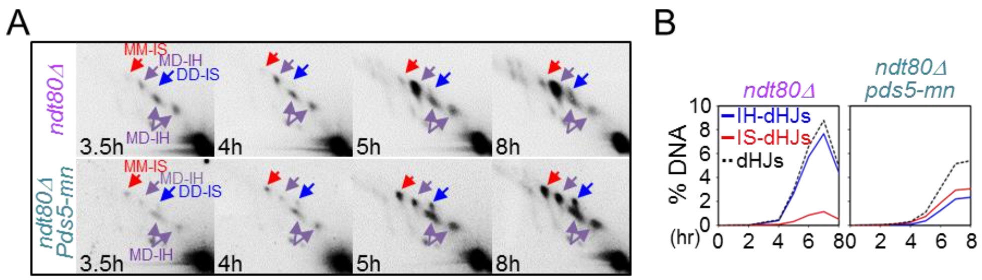

Supplementary Figure S11. 2D gel analysis of IH-SEI formation.

(**A**) 2D gel analysis in *ndt80Δ* and *ndt80Δ pCLB2-PDS5*. (**B**) Quantification of SEIs and dHJs shown in (A).

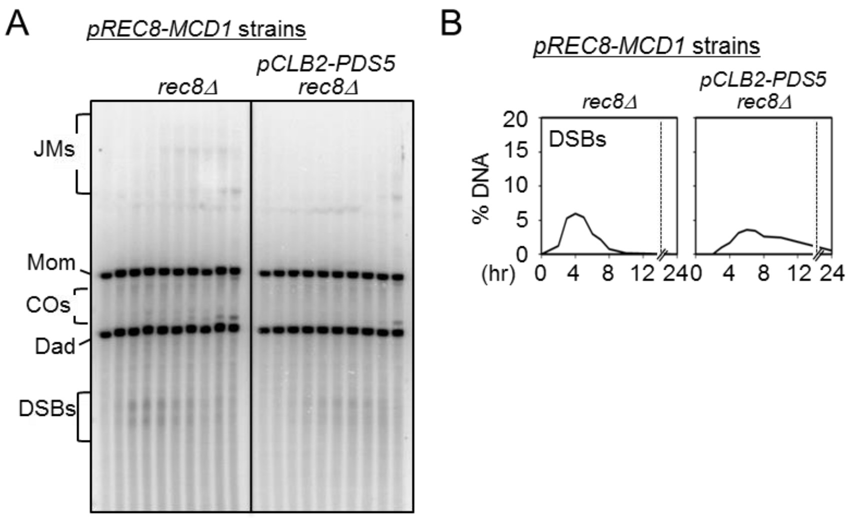

Supplementary Figure S12. DSB formation in *pREC8-MCD1 rec8Δ* and *pREC8-MCD1 pCLB2-PDS5 rec8Δ.*

(**A**) 1D gel analysis at the *HIS4LEU2* locus for in *pREC8-MCD1 rec8Δ* and *pREC8-MCD1 pCLB2-PDS5 rec8Δ* strains. (**B**) Quantification of DSBs shown in (A).

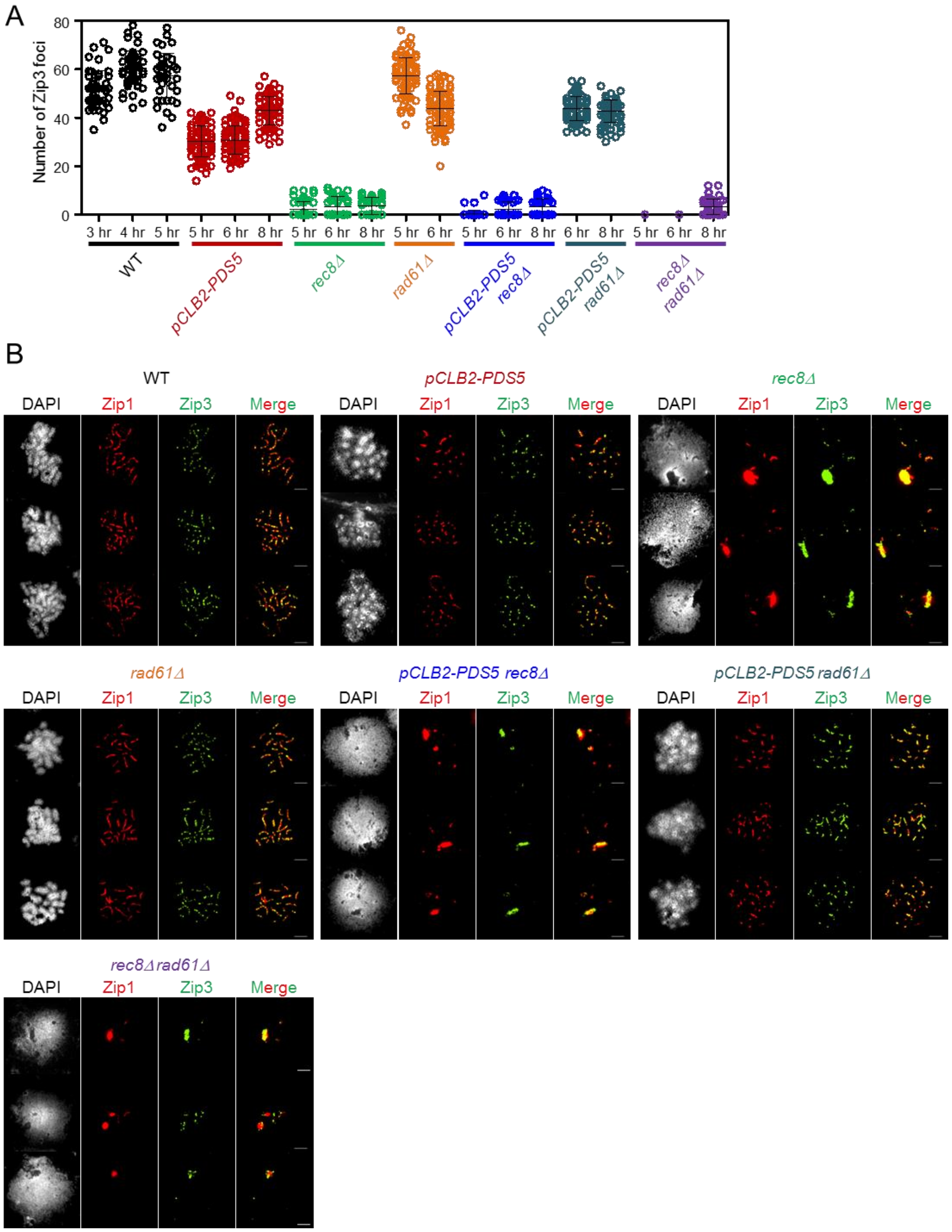

Supplementary Figure S13. Analysis of CO-related Zip3 focus formation in WT, *pCLB2-PDS5*, *rec8Δ*, *rad61Δ*, *pCLB2-PDS5 rec8Δ*, *pCLB2-PDS5 rad61Δ* and *rec8Δ rad61Δ*. (**A**) Quantification of the number of Zip3 focus at pachytene or pachytene-like stage. Error bars indicate mean ± SD (n = 80 ~ 150). (**B**) Representative images showing SC formation and Zip3 foci in mutants. Scale bars represent 2.5 μm.

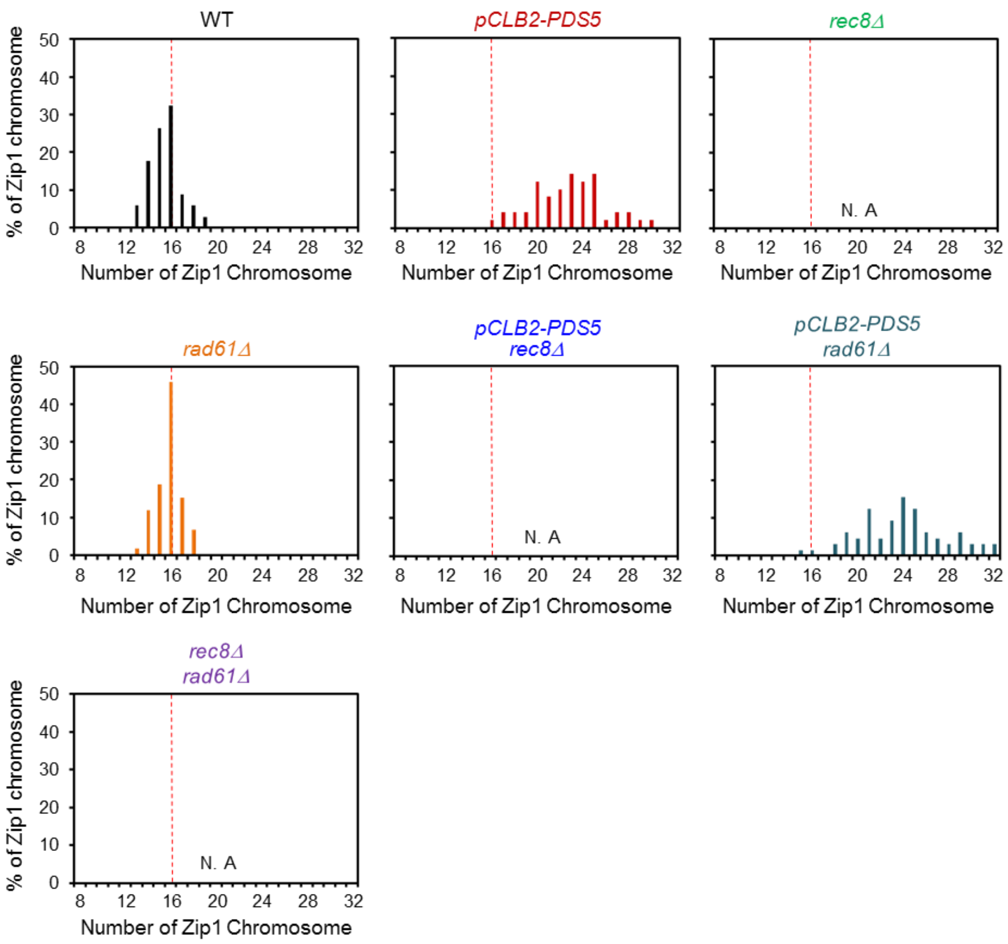

Supplementary Figure S14. Analysis of number of Zip1 chromosome.

Percent of number of Zip1 chromosomes per a cell at pachytene or pachytene-like stage (4 h for WT; 8 h for *pCLB2-PDS5*; 8 h for *rec8Δ*; 5 h for *rad61Δ*; 8 h for *pCLB2-PDS5 rec8Δ*; 8 h for *pCLB2-PDS5 rad61Δ*; 8 h for *rec8Δ rad61Δ*).

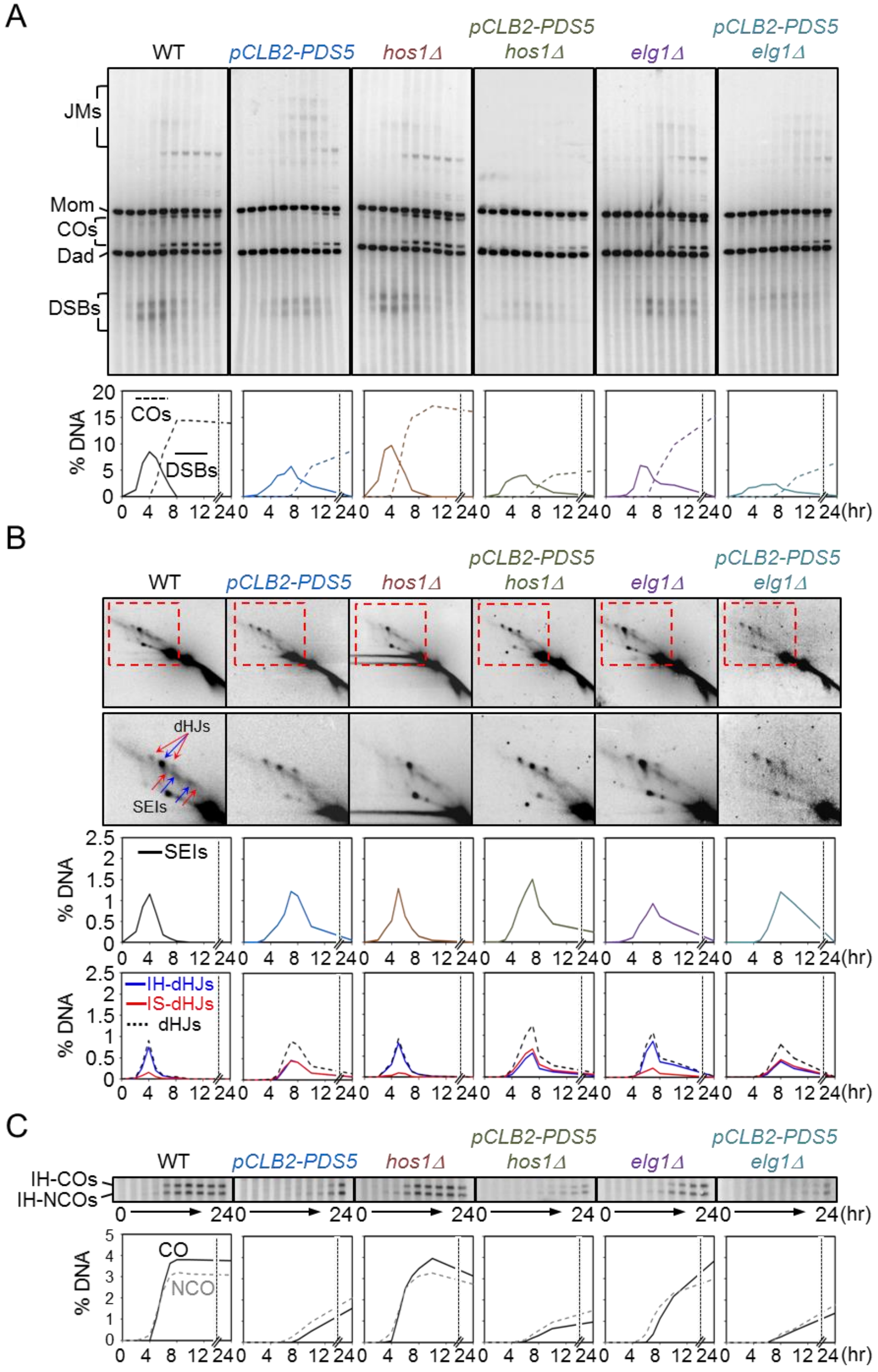

Supplementary Figure S15. Absence of Hos1 and Elg1 does not suppress recombination defect in *pCLB2-PDS5* cells.

(**A**) Analysis of DSBs and COs in WT, *pCLB2-PDS5*, *hos1Δ*, *pCLB2-PDS5 hos1Δ*, *elg1Δ* and *pCLB2-PDS5 elg1Δ*. (**B**) 2D gel analysis. Upper panels show 2D gel images and low panels show JM species as percentage of total hybridizing DNA signals. (**C**) 1D gel analysis of CO and NCO. Quantitative data are shown below each gel image.

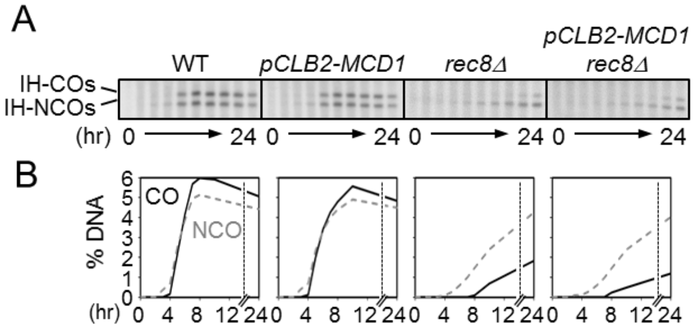

Supplementary Figure S16. Roles of Rec8 and Mcd1 in meiotic recombination. (**A**) Analysis of CO and NCO. (**B**) Quantitative data shown in (A).

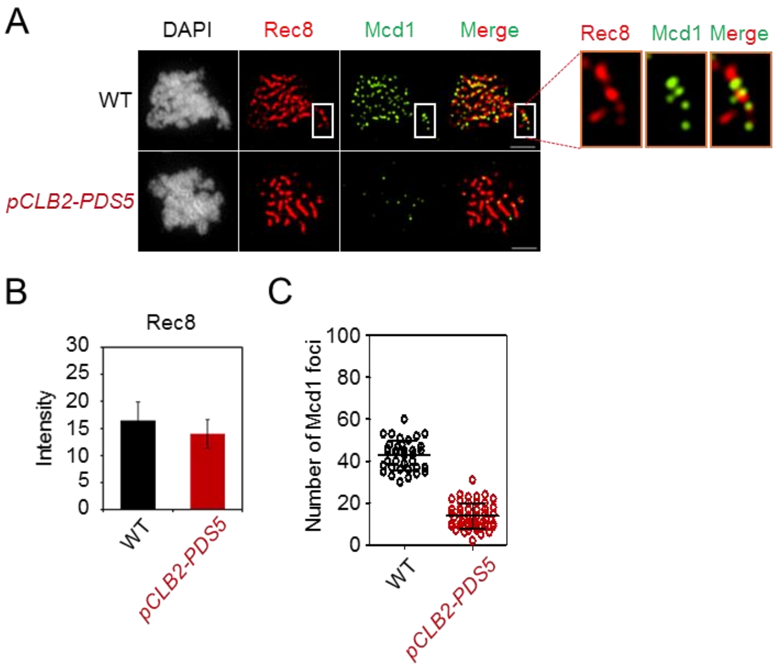

Supplementary Figure S17. Immunofluorescence analysis of localization of Rec8 and Mcd1 on chromosomes.

(**A**) Localization of Rec8 and Mcd1 on pachytene stage in WT and *pCLB2-PDS5*. Spread chromosomes were immunostained for Rec8-3HA and Mcd1-9Myc. Scale bars represent 2.5 µm. (**B**) Quantification of Rec8 intensity on pachytene stage in WT and *pCLB2-PDS5*. Rec8 fluorescence of the whole nucleus was quantified with Image J. The area of a chromosome spread was defined with DAPI staining, and the intensity was quantified within this area. Error bars indicate mean ± SD (n = 34 ~ 50). (**C**) Number of Mcd1 foci on pachytene stage in WT and *pCLB2-PDS5*. The colored scatter plots show the number of Mcd1 foci at pachytene stage (5 h for WT; 8 h for *pCLB2-PDS5*). Error bars indicate mean ± SD (n = 34 ~ 50).
